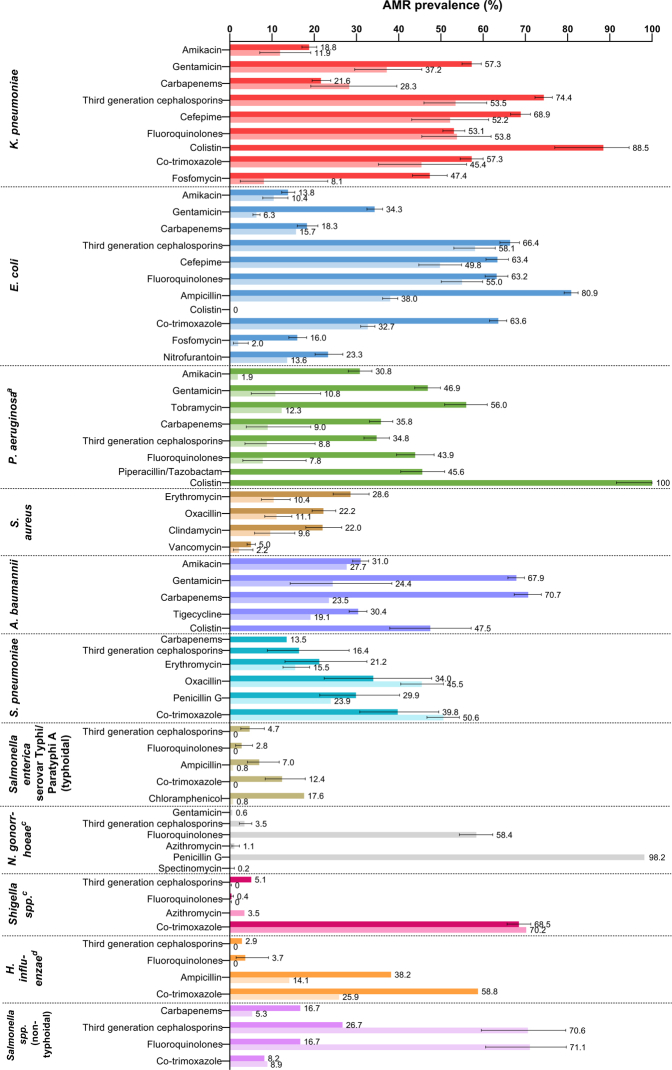# Antimicrobial resistance among common bacterial pathogens in Indonesia: a systematic review

**DOI:** 10.1016/j.lansea.2024.100414

**Published:** 2024-05-13

**Authors:** Michael W. Gach, Gilbert Lazarus, Daniel Martin Simadibrata, Robert Sinto, Yulia Rosa Saharman, Ralalicia Limato, Erni J. Nelwan, H. Rogier van Doorn, Anis Karuniawati, Raph L. Hamers

**Affiliations:** a Oxford University Clinical Research Unit Indonesia, Faculty of Medicine Universitas Indonesia, Jakarta, Indonesia; b Faculty of Medicine, Universitas Indonesia, Jakarta, Indonesia; c Division of Gastroenterology and Hepatology, Mayo Clinic, Rochester, MN, USA; d Centre for Tropical Medicine and Global Health, Nuffield Department of Medicine, University of Oxford, Oxford, UK; e Department of Internal Medicine, Division of Tropical Medicine and Infectious Diseases, Dr. Cipto Mangunkusumo Hospital, Faculty of Medicine Universitas Indonesia, Jakarta, Indonesia; f Department of Clinical Microbiology, Dr. Cipto Mangunkusumo Hospital, Faculty of Medicine Universitas Indonesia, Jakarta, Indonesia; g Oxford University Clinical Research Unit, Hanoi, Vietnam

**Keywords:** Antimicrobial resistance, Systematic review, Antimicrobial susceptibility, Bacteria, GLASS, Indonesia

## Abstract

**Background:**

The WHO Global Antimicrobial Resistance Surveillance System (GLASS) aims to describe antimicrobial resistance (AMR) patterns and trends in common bacterial pathogens, but data remain limited in many low and middle-income countries including Indonesia.

**Methods:**

We systematically searched Embase, PubMed and Global Health Database and three Indonesian databases for original peer-reviewed articles in English and Indonesian, published between January 1, 2000 and May 25, 2023, that reported antimicrobial susceptibility for the 12 GLASS target pathogens from human samples. Pooled AMR prevalence estimates were calculated for relevant pathogen-antimicrobial combinations accounting for the sampling weights of the studies (PROSPERO: CRD42019155379).

**Findings:**

Of 2182 search hits, we included 102 papers, comprising 19,517 bacterial isolates from hospitals (13,647) and communities (5870). In hospital settings, 21.6% of *Klebsiella pneumoniae* isolates, 18.3% of *Escherichia coli* isolates, 35.8% of *Pseudomonas aeruginosa* isolates and 70.7% of *Acinetobacter baumannii* isolates were carbapenem-resistant; 29.9% of *Streptococcus pneumoniae* isolates were penicillin-resistant; and 22.2% of *Staphylococcus aureus* isolates were methicillin-resistant. Hospital prevalence of carbapenem-resistant *K. pneumoniae* and *E. coli*, and penicillin-resistant *S. pneumoniae* increased over time. In communities, 28.3% of *K. pneumoniae* isolates and 15.7% of *E. coli* isolates were carbapenem-resistant, 23.9% of *S. pneumoniae* isolates were penicillin-resistant, and 11.1% of *S. aureus* isolates were methicillin-resistant. Data were limited for the other pathogens.

**Interpretation:**

AMR prevalence estimates were high for critical gram-negative bacteria. However, data were insufficient to draw robust conclusions about the full contemporary AMR situation in Indonesia. Implementation of national AMR surveillance is a priority to address these gaps and inform context-specific interventions.

**Funding:**

10.13039/100004440Wellcome Africa Asia Programme Vietnam.

**Research in context**

**Evidence before this study**

We reviewed situation analysis reports, the WHO Global Antimicrobial Resistance Surveillance System (GLASS) website, and searched PubMed with the terms “Indonesia”, “antibiotic or antimicrobial resistance”, and “human health”, until November 1st 2023, in English and Indonesian language. Indonesia is a potential hotspot for antimicrobial resistance (AMR) because of high infectious disease burdens, coupled with weakly enforced antibiotic regulations in human and veterinary health, and other health system vulnerabilities. Two recent reports aggregated routine culture data from sentinel hospitals (GLASS submission, 20 hospitals, and Indonesian Society for Clinical Microbiology, 70 hospitals, both from 2022), describing high prevalence estimates for third-generation cephalosporin (3GC) (53–79%) and carbapenem (29–30%) resistant *Klebsiella pneumoniae;* 3GC (66–70%) and carbapenem (15–15%) resistant *Escherichia coli;* carbapenem-resistant *Acinetobacter* spp. (76–89%); methicillin-resistant *Staphylococcus aureus* (38–40%); ceftazidime (41%) and carbapenem (45%) resistant *Pseudomonas aeruginosa*; and penicillin-resistant *Streptococcus pneumoniae* (20%) (Panel). A comprehensive review on AMR in human health in Indonesia has not been conducted to date.

**Panel** **A comparison between Indonesia, other Southeast Asian countries and the European Union of AMR prevalence estimates for key pathogen-antimicrobial combinations in hospitalised populations.**

**Table:** 

	Indonesia			Malaysia	Philippines	Thailand	Vietnam	European Union
Country income level^a^	Upper-middle			Upper-middle	Lower-middle	Upper-middle	Lower-middle	High income
Population size (in million)^b^	275.5			33.9	115.6	71.7	98.2	448.0
AMR data source^c^	Systematic review (2000–2023)	GLASS (2021)^8^	Indonesian society for clinical microbiology (PAMKI) report (2022)^10^	National antibiotic resistance surveillance report (2021)^19^	AMR surveillance program report (2022)^21^	GLASS (2021)^8^	VINARES network (2016–2017)^20^	ECDC report (2021)^22^
3GC-resistant *E. coli*	66%	70%	66%	21%	42%	35%	66%	14%
Carbapenem-resistant *E. coli*	18%	15%	15%	1%	9%	1.6%	11%	0.2%
3GC-resistant *K. pneumoniae*	74%	79%	53%	25%	46%	39%	53%	34%
Carbapenem-resistant *K. pneumoniae*	22%	17%	29%	6%	16%	12%	27%	12%
Ceftazidime-resistant *P. aeruginosa*	35%	NS	41%	8%	15%	17%	46%	16%
Carbapenem-resistant *P. aeruginosa*	36%	NS	45%	9%	15%	18%	45%	18%
Carbapenem-resistant *A. baumannii*	71%	89%	76%	69%	53%	49%	79%	40%
Penicillin-resistant *S. pneumoniae*	30%	20%	NS	3%	16%	38%	58%	16%
Methicillin-resistant *S. aureus*	22%	40%	38%	7%	33%	25%	73%	16%

3GC, third-generation cephalosporins; ECDC, European Centre for Disease Prevention and Control; GLASS, WHO Global Antimicrobial Resistance Surveillance System; NS, not specified; VINARES, Viet Nam Resistance.

a World Bank Group country classification by income level 2022.

b Data from World Bank group (https://data.worldbank.org/indicator/SP.POP.TOTL?locations=MY-TH-VN-PH-EU-ID&name_desc=false).

c Data shown in table are based on the most recent available data sources.

**Added value of this study**

This review represents an aggregation of the peer-reviewed literature on the magnitude and patterns of AMR in common bacterial pathogens most relevant to human health in Indonesia, including nearly 20,000 isolates from 102 studies spanning the past 23 years. This countrywide evidence synthesis can be a guiding tool for policymakers and medical practitioners. Hospital-based levels of AMR to important antibiotics in common gram-negative bacteria were estimated to be among the highest reported in the Southeast Asia region. These included carbapenem-resistant *Acinetobacter baumannii*, *Pseudomonas* *aeruginosa,* *Escherichia coli* and *Klebsiella pneumoniae*, all classified as Priority 1 (“critical”) bacteria on the WHO bacterial priority pathogen list for research and development of new antibiotics. The review identified areas where critical information is lacking, particularly on specific bacterial pathogens, community settings, geographic settings outside of Java island, as well as clinical metadata for classification of isolates.

**Implications of all the available evidence**

The management of severe infections associated with gram-negative bacteria in Indonesia is increasingly dependent on more expensive and less readily available antibiotics. Accelerated implementation of national AMR surveillance and the National Action Plan for AMR containment is an urgent priority.

## Introduction

Antimicrobial resistance (AMR) is a global public health priority with significant economic consequences and a disproportionate impact in low and middle-income countries (LMICs).^1^ Antibiotic-resistant bacterial infections have been estimated to be associated with 4.95 million deaths worldwide in 2019 alone.^2^ Southeast Asia has been identified as a region of great importance in the development and spread of AMR, driven by overuse and misuse of antimicrobial agents, poor sanitation, hygiene and infection control, and other health system vulnerabilities to AMR.^3^^,^^4^ In studies from Thailand,^5^ Cambodia,^6^ and Indonesia,^7^ community and hospital-acquired drug-resistant bacteraemias have been associated with increased mortality and hospital costs.

National and global AMR surveillance systems are important to provide evidence for empiric treatment guidelines, estimate epidemiological burdens across time and space, and assess the impact of public health policies and interventions. To mitigate the impact of AMR, in 2015 the World Health Organization (WHO) coordinated the development of the Global Antimicrobial Resistance Surveillance System (GLASS), as a component of the Global Action Plan on AMR.^8^ However, despite progress, many LMICs have not been able to aggregate data to provide a representative country-level perspective.

Indonesia is a vast archipelagic nation with the world’s fourth largest population (275 million), stark socio-economic and health inequalities, and a decentralised healthcare system with weakly enforced antibiotic regulations.^9^ Limited available data have suggested high levels of AMR against key antibiotics for common clinically important, especially gram-negative, bacterial pathogens in hospital populations,^8^^,^^10^ and an estimated 34,530–133,753 deaths because of bacterial AMR in 2019.^11^ This systematic review aimed to aggregate all available data in the human health sector published in the peer-reviewed literature in Indonesia since the year 2000, to estimate AMR prevalence for relevant pathogen-antimicrobial combinations, focusing on the twelve GLASS target bacterial pathogens, i.e. *Acinetobacter* *baumannii, Escherichia* *coli, Haemophilus influenzae, Klebsiella* *pneumoniae, Neisseria gonorrhoeae, Neisseria meningitidis, Pseudomonas* *aeruginosa, Salmonella enterica* serovar Typhi and Paratyphi A (typhoidal), *Salmonella* spp. (non-typhoidal), *Shigella spp., Staphylococcus* *aureus,* and *Streptococcus pneumoniae*, both for hospital and community settings.^12^

## Methods

### Search strategy and selection criteria

This systematic review was reported according to the Preferred Reporting Items for Systematic Reviews and Meta-analyses (PRISMA) 2020 guidelines. The protocol was registered in PROSPERO (CRD42019155379). We searched three international (PubMed, Embase and Global Health Database) and three Indonesian (*Garba Rujukan Digital*, *Neliti* and *Jurnal Penelitian dan Pengembangan Pelayanan Kesehatan*) bibliographic databases for eligible articles, published since January 1, 2000 until May 25, 2023. The searches combined the terms “Indonesia”, “antimicrobial or antibiotic resistance”, “susceptibility”, “resistance”, plus each of the bacterial pathogens included in the GLASS-AMR Manual 2.0 (Supplementary Table S1),^12^ restricted to original, peer-reviewed articles in English or Indonesian language. Reports were included if they reported bacterial antibiotic susceptibility testing (AST) on isolates from human specimens collected in Indonesia, regardless of clinical context, and reporting on at least one GLASS bacterial pathogen. Reports were excluded if they were written as reviews, editorials, conference proceedings or individual case reports; completed data collection before 2000; did not report phenotypic AST data for at least one GLASS pathogen-antimicrobial combination or addressed only non-GLASS organisms; AST data had missing numerators/denominators that could not be calculated from raw data; there were fewer than 10 isolates tested for AST; addressed only samples from non-human sources; or Indonesian and non-Indonesian data could not be separated.

### Article screening and selection

Two independent reviewers (MG, GL, DS, RS) screened titles and abstracts of all articles and manually removed duplicate articles and/or datasets. Full-text articles were independently judged for relevance and quality by at least two reviewers (MG, GL, DS, RS). Any disagreements were resolved by a senior researcher (RLH).

### Risk of bias and quality assessment

Because the included studies were neither randomised controlled trials nor comparative studies, traditional methods for assessment of risk of bias were not applicable. Instead, we excluded studies that did not report on an essential set of four core items from the STrengthening the Reporting of Observational studies in Epidemiology (STROBE) checklist (Supplementary Table S2). We additionally recorded the reporting for the 13 mandatory items of the Microbiology Investigation Criteria for Reporting Objectively (MICRO) checklist (Supplementary Table S3).^13^

### Data extraction and synthesis

Data were extracted by two reviewers (MG, GL, DS, RS) onto a predefined extraction form. We extracted and tabulated data on general article information (first author, year of publication), and author-reported summary estimates (not individual-level data) of the AST results for relevant pathogen-antimicrobial combinations (GLASS and additional clinically relevant combinations) (Supplementary Table S4), specimen type (e.g. blood, urine, stool), study population (health status, age group, sex), health care setting (hospital or community), geographic location, period of data collection, and laboratory method information. Studies in communities, primary care, and outpatient clinics were classified as community setting. Studies among inpatients, as well as studies among mixed or unspecified inpatients and outpatients, were classified as hospital setting. For hospital-based studies, as per GLASS, hospital-acquired infection (HAI) was defined as samples taken >2 calendar days after date of admission, and community-acquired infection (CAI) was defined as samples taken ≤2 calendar days after date of admission.

### Ascertainment of AMR

Given that most studies did not report minimal inhibitory concentrations (MICs) for antimicrobials, we used author-reported AST interpretations into susceptible, intermediate or resistant. Intermediate susceptibility, where reported, was considered resistant. Resistance proportions were calculated as the inverse of susceptibility where applicable. AST interpretative criteria used were extracted (e.g. Clinical and Laboratory Standards Institute [CLSI], European Committee on Antimicrobial Susceptibility Testing [EUCAST]). Duplicate or sequential isolates, when reported, were removed from the analysis. Where AST results were given for more than one same-class antibiotic (e.g. third-generation cephalosporins [3GC], fluoroquinolones, group 2 carbapenems [doripenem, imipenem, meropenem]), the highest individual-antibiotic resistance proportion was selected to represent class resistance, except for the aminoglycosides since gentamicin, amikacin and tobramycin show markedly different resistance patterns.^12^ Resistance of *S. aureus* to cefoxitin, methicillin and/or oxacillin were considered methicillin-resistant (MRSA).

### Statistical analysis

Because of substantial heterogeneity in study design, populations studied, period of data collection, and type of specimens collected, we considered quantitative meta-analysis inappropriate.^14^ Instead, pooled AMR prevalence estimates were calculated for relevant pathogen-antimicrobial combinations, as the number of patients with infection caused by pathogen_x_ resistant to antibiotic_y_, divided by total number of patients with infection caused by pathogen_x_ with AST results for antibiotic_y_ (either susceptible, intermediate or resistant), and expressed as percentages,^12^ accounting for the sampling weights (number of isolates) of the individual studies, separately for hospital and community settings. We calculated 95% confidence intervals using the Wilson score interval when the number of studies for a pathogen-antimicrobial combination was more than one. Additionally, we calculated AMR prevalence for pathogen-antimicrobial combinations per five-year time periods from 2000 to 2023, where sufficient data were available. Data management was performed using Microsoft Excel® (Microsoft Corp., Redmond, WA) and visualisations using GraphPad Prism version 9.5.0 (GraphPad Software, Boston, MA).

### Role of the funding resource

The funder of the study had no role in study design, data collection, data analysis, data interpretation, or writing of the report. The first and corresponding authors had full access to all the data and the final responsibility to submit for publication.

## Results

### Study characteristics

**Fig. 1:**
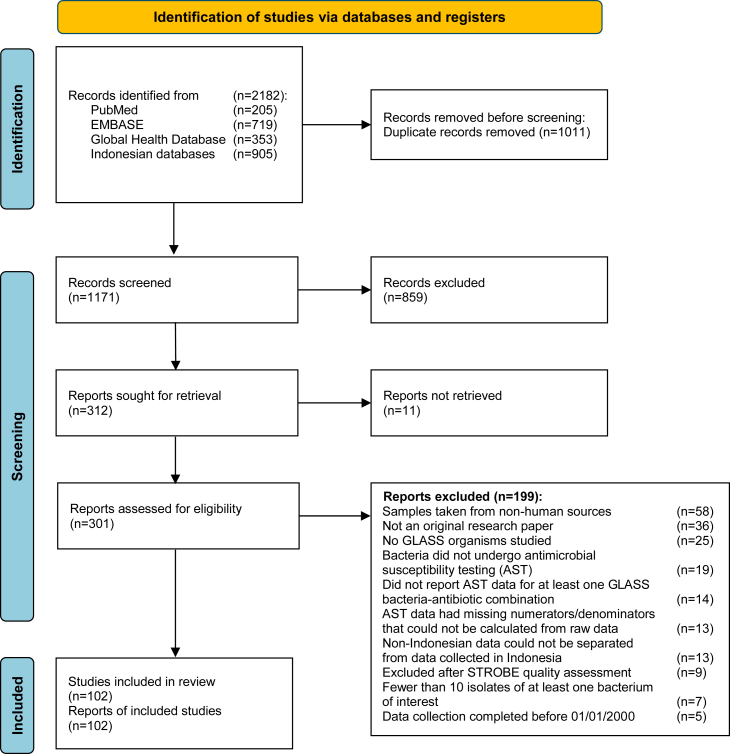
PRISMA flow diagram.

**Fig. 2:**
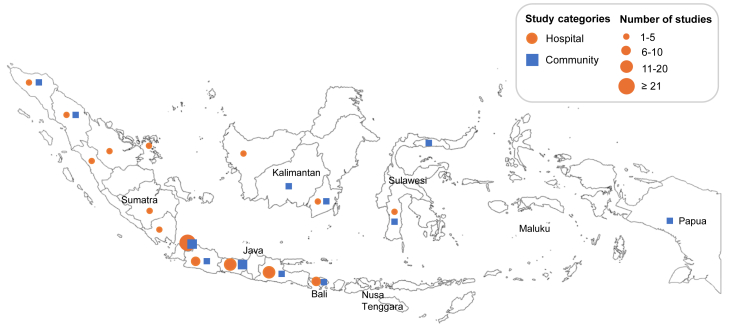
Geographical map of the 102 reports included in the review. The map includes 10 studies that were conducted in multiple provinces (4 in hospitals, 3 in communities, and 3 in both).

The search strategy collectively gave 2182 hits, 205 from PubMed, 353 from Global Health Database, 719 from EMBASE, and 905 from the Indonesian databases (Fig. 1). After duplicate removal (1011) and title and abstract screening, 312 articles remained. After full-text screening and quality assessment, 102 reports were included. Study characteristics are summarized in Table 1 and Supplementary Table S5. 73 (71.6%) reports were conducted in hospital settings, 24 (23.5%) in community settings, and five (4.9%) in both. Of the 73 hospital-based reports, only nine specified isolates associated with HAIs and three with CAIs. Most reports originated from Java island (75.5%, 77/102) (Fig. 2). 37 reports (36.3%) included only adults, 25 (24.5%) both adults and children, 19 (18.6%) only children, and 21 (20.6%) did not specify age groups.

**Table 1:** Characteristics of included reports.

Total reports	N = 102	%
**GLASS bacterial pathogens**		
*Klebsiella pneumoniae*	42	41.2%
*Escherichia coli*	41	40.2%
*Pseudomonas aeruginosa*	32	31.4%
*Staphylococcus aureus*	28	27.5%
*Acinetobacter baumannii*	27	26.5%
*Streptococcus pneumoniae*	18	17.6%
*Salmonella enterica* serovar Typhi/Paratyphi A	7	6.9%
*Neisseria gonorrhoeae*	6	5.9%
*Shigella* spp.	5	4.9%
*Haemophilus influenzae*	3	2.9%
*Salmonella* spp.	3	2.9%
*Neisseria meningitidis*	0	0.0%
**Specimen type**		
Blood	33	32.4%
Lower respiratory tract	30	29.4%
Urine	26	25.5%
Stool/rectal swab	9	8.8%
Urethral, cervical, rectal, pharyngeal swabs	6	5.9%
Cerebrospinal fluid	4	3.9%
Other^a^	48	47.1%
**Phenotypic AST method**		
Diffusion methods	60	58.8%
Automated system (Phoenix, Vitek)	28	27.5%
Dilution methods (micro-, macro-, agar dilution)	13	12.7%
MALDI-TOF MS	5	4.9%
E-test	3	2.9%
Chromogenic agar	1	1.0%
Not specified	15	14.7%
**AST interpretative criteria**		
CLSI	61	59.8%
EUCAST	9	9.1%
Not specified	32	32.3%
**Study design**		
Cross-sectional	51	50.0%
Retrospective	39	38.2%
Prospective	12	11.8%
**Geographical location**^b^		
Java	77	75.5%
Sumatra	19	18.6%
Bali	9	8.8%
Kalimantan	6	5.9%
Sulawesi	4	3.9%
Papua	1	1.0%
**Study setting**		
Hospitals	73	71.6%
Communities	24	23.5%
Mixed	5	4.9%
**Year of publication**		
2020–2023	38	37.3%
2015–2019	31	30.4%
2010–2014	21	20.6%
2005–2009	9	8.8%
2000–2004	3	2.9%
**Patient age groups**		
Adults	37	36.3%
Adults and children	25	24.5%
Children	19	18.6%
Not specified	21	20.6%
**Patient sex**		
Females and males	60	58.8%
Female only	5	4.9%
Not specified	37	36.3%

Total N for some characteristics is greater than N = 102 because some reports were included in more than category, i.e. for GLASS bacterial pathogens (44 reports), specimen type (24), phenotypic AST method (19), geographic location (7), and AST interpretative criteria (3).

AST, antimicrobial susceptibility testing; CLSI, Clinical and Laboratory Standards Institute; EUCAST, European Committee on Antimicrobial Susceptibility Testing; GLASS, Global Antimicrobial Resistance and Use Surveillance System; MALDI-TOF MS, matrix-assisted laser desorption/ionization coupled with time-of-flight mass spectrometry.

a Other specimen types include pus (16 studies, 15.7%), nasopharyngeal swabs (13 studies, 12.7%), wound/ulcer swabs (11 studies, 10.8%), throat swab (6 studies, 5.9%), tissue samples (5 studies, 4.9%), nasal swabs (3 studies, 2.9%), pleural fluid (3 studies, 2.9%), middle ear fluid (1 study, 1.0%), ear swab (1 study, 1.0%), vaginal swab (1 study, 1.0%), other body fluid (1 study, 1.0%) and unspecified (2 study, 2.0%). One study may list multiple other specimen types.

b There were no included reports originating from Maluku or Nusa Tenggara.

**Fig. 3:**
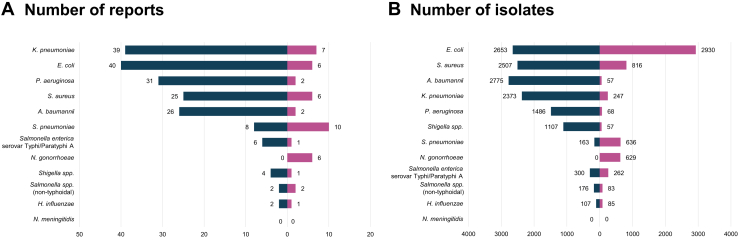
Number of reports and bacterial isolates included for each GLASS bacterial pathogen. Figure shows tornado plots of (A) the number of included reports that described resistance data for each of the GLASS bacterial pathogens (102 overall; 73 from hospitals, 24 from communities, 5 from both); and (B) the number of included bacterial isolates for each of the GLASS bacterial pathogens (19,517 overall; 13,647 from hospitals and 5870 from communities), stratified by hospitals (blue) and communities (purple). Data are sorted in descending order of number of total reports and isolates in hospitals and communities.

The total number of bacterial isolates included in the review was 19,517, comprising 13,647 from hospitals and 5870 from community settings (GLASS priority specimen types are summarised in Supplementary Table S6). The most commonly reported bacterial pathogens were *K. pneumoniae* (42 reports, 2620 isolates), followed by *E. coli* (41 reports, 5583 isolates), *P. aeruginosa* (32 reports, 1554 isolates), *S. aureus* (28 reports, 3323 isolates), *A. baumannii* (27 reports, 2832 isolates), *S. pneumoniae* (18 reports, 799 isolates), *S. enterica* serovar Typhi and Paratyphi A (7 reports, 562 isolates), *N. gonorrhoeae* (6 reports, 629 isolates), *Shigella spp* (5 reports, 1164 isolates), *Salmonella spp* (non-typhoidal) (3 reports, 259 isolates), and *H. influenzae* (3 reports, 192 isolates). There were no reports describing *N. meningitidis* (Fig. 3). AST was mostly performed by disc diffusion method (60 reports), followed by automated systems (mostly VITEK; 28 reports), dilution methods (mostly microdilution; 13 reports), MALDI-TOF (5 reports), E-test (3 reports), chromogenic agar (1 study), or unspecified (15 reports). Minimum inhibitory concentrations were only reported in 15 reports (14.7%). AST interpretative criteria used were stated in 67 reports (65.7%), including CLSI (61 reports) and EUCAST (9 reports) (13 reports did not report the specific edition used). Genotypic methods were additionally used for confirmatory testing in 23 reports, including PCR (19 reports), multilocus sequence typing (4 reports), and next-generation sequencing (1 report).

Of the 13 MICRO mandatory items, one report (0.98%) described all items, 25 reports (24.5%) missed 1–2, 56 (54.9%) missed 3–4 and 20 (19.6%) missed 5 or more items, with a median of 3 (range 0–8) missing items. The most frequently missing mandatory items were external QA (item #12, 98 reports, 96.1%), duplicate and sequential isolates (item #14, 84 reports, 82.4%) and AMR definitions (item #11, 70 reports, 68.6%) (Supplementary Table S3).

### Proportions of resistance for each of the pathogen-antimicrobial combinations

#### Klebsiella pneumoniae

42 reports described 2620 *K. pneumoniae* isolates, including 39 hospital reports (2373 isolates) and 7 community reports (247 isolates).

**Fig. 4:**
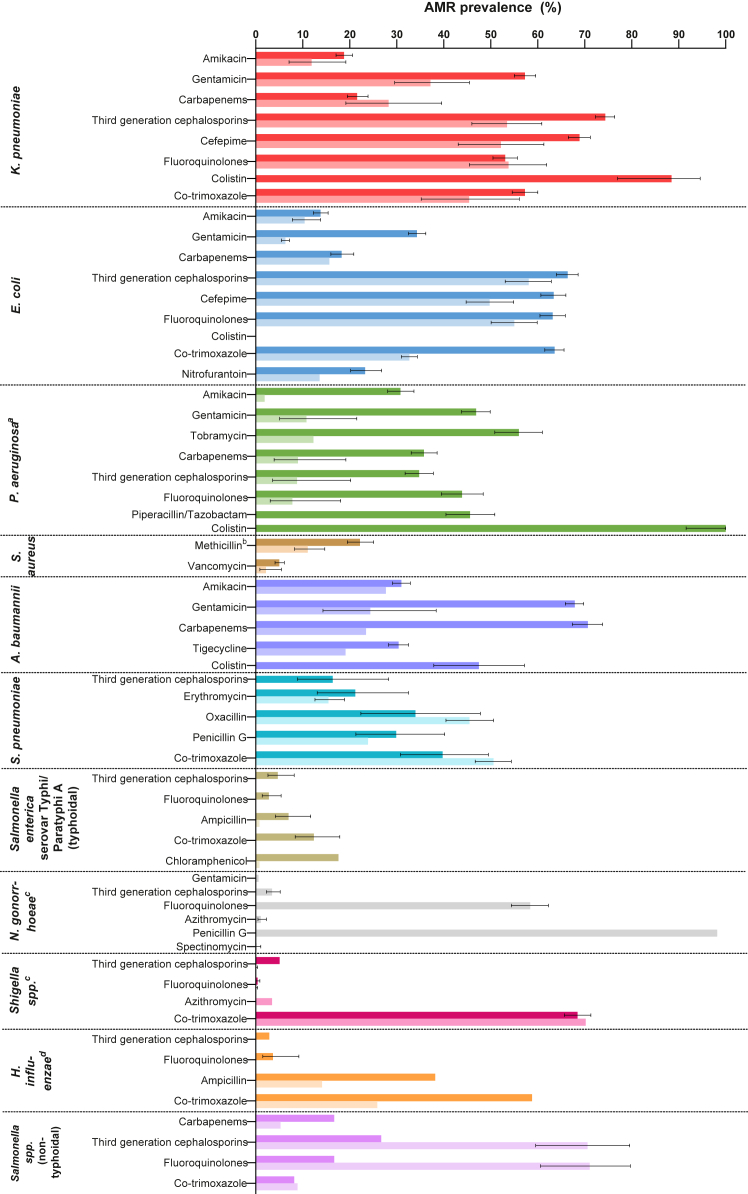
AMR prevalence estimates for GLASS-specific pathogen-antimicrobial combinations in hospital and community settings. Figure shows bar charts of AMR prevalence estimates for GLASS-specific pathogen-antimicrobial combinations, for hospitals (dark color) and communities (light color), accounting for sampling weights of the individual studies (see Supplementary Table S4 for further details). 95% confidence intervals were estimated using Wilson score interval (where the number of studies was greater than one). ^a^The estimate for third-generation cephalosporins is derived only from ceftazidime. ^b^Resistance to methicillin detected using either cefoxitin or oxacillin. ^c^The estimate for fluoroquinolones is derived only from ciprofloxacin. ^d^The estimate for fluoroquinolones is derived only from levofloxacin.

**Fig. 5:**
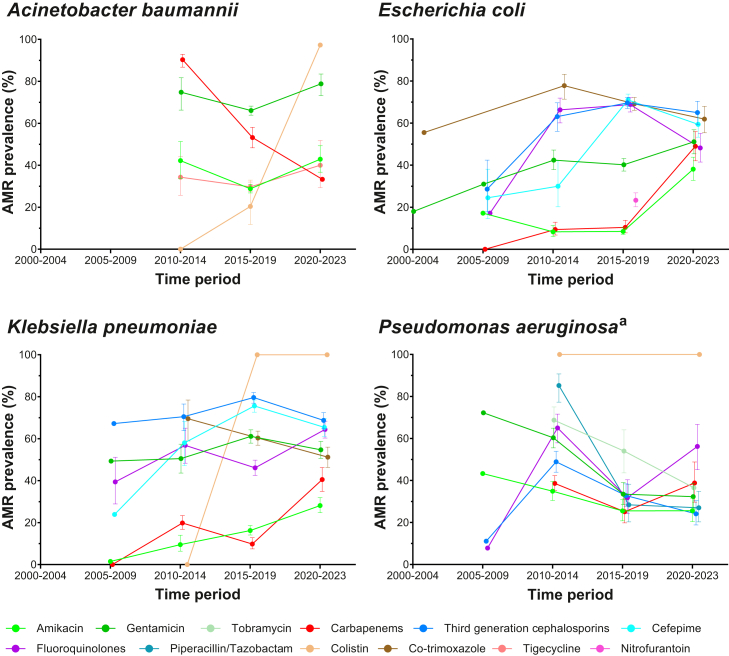
AMR prevalence for pathogen-antimicrobial combinations in selected gram-negative GLASS pathogens in hospitals per 5-year time periods. Figure shows AMR prevalence estimates per 5-year time periods for GLASS-specific pathogen-antimicrobial combinations per time period in selected gram-negative GLASS pathogens in hospitals, accounting for sampling weights of the individual studies (see Supplementary Table S4 for further details). The dots were connected by lines to improve interpretation of the data. Pathogens for which insufficient data were available are not shown. For studies that collected data for two years, the latest year was used as the year of data collection. For studies that collected data for more than two years, the median of the period of data collection (rounded to the nearest integer) was used as the year of data collection. 95% confidence intervals were estimated using Wilson score interval (where the number of studies was greater than one). ^a^The estimate for third-generation cephalosporins is derived only from ceftazidime.

In hospitals, the percentage of *K. pneumoniae* isolates exhibiting resistance against 3GC was 74.4% (95% CI 72.3–76.4; 1345/1800); carbapenems 21.6% (19.5–23.9; 409/2029); fluoroquinolones 53.1% (50.5–55.7; 783/1461); cefepime 68.9% (66.5–71.2; 995/1473); gentamicin 57.3% (55.0–59.6; 1003/1771); amikacin 18.8% (17.1–20.6; 336/1952); co-trimoxazole 57.3% (54.6–60.0; 767/1299); fosfomycin 47.4% (43.3–51.5; 307/575); and colistin 88.5% (77.0–94.6; 46/52) (Fig. 4, Supplementary Fig. S1 and Table S7). Across the ten hospital reports that distinguished HAI and CAI, the percentage of *K. pneumoniae* isolates exhibiting resistance against 3GC was 85.7% (82.6–88.3; 401/472) and 48.5% (37.1–60.2; 33/68); carbapenems 32.6% (28.7–36.7; 257/306) and 29.6% (8/27); fluoroquinolones 70.9% (66.1–75.3; 280/395) and 70.4% (19/27); cefepime 83.6% (80.3–86.4; 257/306) and unknown (no CAI data); gentamicin 66.5% (62.5–70.3; 343/520) and 18.5% (5/27); amikacin 36.4% (32.5–40.4; 191/528) and 7.4% (19/27); and co-trimoxazole 52.3% (48.1–56.4; 134/246) and 22.2% (6/27), respectively. Compared to previous years (2015–2019), recent (2000–2023) percentages of *K. pneumoniae* isolates exhibiting resistance were higher for carbapenems (40.5% [34.9–46.2] vs 9.8% [7.5–12.8]), fluoroquinolones (64.4% [60.3–68.3] vs 46.1% [42.5–49.8]) and amikacin (28.1% [24.7–31.9] vs 16.2% [14.1–18.6%]); and lower for 3GC (68.7% [64.8–72.4] vs 79.6% [76.9–82.0]), cefepime (65.4% [61.1–69.6] vs 75.6% [72.6–78.4]), gentamicin (54.7% [50.6–58.7] vs 61.1% [57.9–64.2]), and co-trimoxazole (51.2% [46.3–56.0] vs 60.3% [56.9–63.6]) (Fig. 5).

In communities, the percentage of *K. pneumoniae* isolates exhibiting resistance against 3GC was 53.5% (95% CI 46.0–60.9; 90/168); carbapenems 28.3% (19.2–39.6; 20/72); fluoroquinolones 53.8% (45.5–61.9; 73/139); cefepime 52.2% (43.1–61.3; 57/112); gentamicin 37.2% (29.6–45.5; 50/138); amikacin 11.9% (7.1–19.2; 17/112); co-trimoxazole 45.4% (35.2–56.1; 28/83); and fosfomycin 8.1% (2.6–23.2; 3/30).

Across the eight studies that reported on the resistance mechanisms for *K. pneumoniae*, the percentage of extended-spectrum beta-lactamase (ESBL) producing isolates was 67.4% (95% CI 63.7–71.0; 426/632).

#### Escherichia coli

41 reports described 5583 *E. coli* isolates, including 40 hospital reports (2653 isolates) and 6 community reports (2930 isolates).

In hospitals, the percentage of *E. coli* isolates exhibiting resistance against 3GC was 66.4% (95% CI 64.0–68.6; 1059/1625); carbapenems 18.3% (16.0–20.9; 181/976); fluoroquinolones 63.3% (60.5–65.9; 754/1191); cefepime 63.4% (60.7–66.0; 760/1272); gentamicin 34.3% (32.5–36.2; 865/2539); amikacin 13.8% (12.3–15.4; 260/1949); ampicillin 80.9% (79.2–82.5; 1710/2105); co-trimoxazole 63.6% (61.5–65.6; 1289/2062); nitrofurantoin 23.3% (20.2–26.8; 125/630); fosfomycin 16.0% (14.0–18.2; 155/1138) and colistin 0.0% (0/4) (Fig. 4, Supplementary Fig. S1 and Table S8). Across the six hospital reports that distinguished HAI and CAI, the percentage of *E. coli* isolates exhibiting resistance against 3GC was 76.1% (70.9–80.6; 204/270) and 30.0% (3/10); carbapenems 13.9% (10.1–19.0; 24/170) and 20.0% (2/10); fluoroquinolones 70.6% (65.2–75.5; 139/210) and 40.0% (4/10); cefepime 77.7% (72.0–82.6; 80/107) and unknown (no CAI data); gentamicin 43.7% (37.5–50.1; 94/215) and 10.0% (1/10); amikacin 10.9% (7.9–15.0; 30/284) and 20.0% (2/10); ampicillin 96.6% (85/88) and unknown (no CAI data); co-trimoxazole 68.1% (62.0–73.7; 62/92) and 30.0% (3/10); and fosfomycin 6.3% (4/64) and unknown (no CAI data) respectively. Compared to previous years (2015–2019), recent (2000–2023) percentages of *E. coli* isolates exhibiting resistance were higher for carbapenems (49.0% [42.1–56.0] vs 10.4% [7.8–13.8]), gentamicin (51.1% [45.4–56.8] vs 40.2% [37.2–43.2]), and amikacin (38.1% [32.8–43.8] vs 8.5% [7.0–10.2]); lower for fluoroquinolones (48.2% [41.5–55.1] vs 68.8% [65.3–72.1]), cefepime (59.4% [53.3–65.2] vs 71.0% [67.9–73.8]), and ampicillin (23.5% [16.4–32.6] vs 93.6% [91.8–95.0]); and were stable for 3GC (65.0% [59.3–70.4] vs 69.6% [66.9–72.3]) and co-trimoxazole (61.9% [55.4–68.0] vs 69.2% [66.1–72.2]) (Fig. 5).

In communities, the percentage of *E. coli* isolates exhibiting resistance against 3GC was 58.1% (95% CI 53.1–62.9; 222/385); carbapenems 15.7% (13/83); cefepime 49.8% (44.8–54.9; 181/377); fluoroquinolones 55.0% (50.1–69.9, 220/396); gentamicin 6.3% (5.5–7.2; 181/2913); amikacin 10.4% (7.8–13.8; 41/397); ampicillin 38.0% (36.2–39.8; 1109/2813); co-trimoxazole 32.7% (31.0–34.4; 913/2854); nitrofurantoin 13.6% (38/280); and fosfomycin 2.0% (0.9–4.4; 6/294).

Across the six studies that reported on the resistance mechanisms for *E. coli*, the percentage of ESBL-producing isolates was 60.1% (95% CI 56.3–63.7; 405/674).

#### Pseudomonas aeruginosa

32 reports described 1554 *P. aeruginosa* isolates, including 31 hospital reports (1486 isolates) and 2 community reports (68 isolates).

In hospitals, the percentage of *P. aeruginosa* isolates exhibiting resistance against ceftazidime was 34.8% (95% CI 31.8–37.8; 340/972); piperacillin/tazobactam 45.6% (40.5–50.9; 157/344); carbapenems 35.8% (33.1–38.6; 407/1136); fluoroquinolones 43.9 (39.5–48.4%; 210/478); gentamicin 46.9% (43.8–49.9; 481/1030); amikacin 30.8% (28.1–33.7; 319/1038), tobramycin 56.0% (50.9–61.0; 202/363); and colistin 100% (91.6–100.0; 42/42) (Fig. 4, Supplementary Fig. S1 and Table S9). Across the six hospital reports that distinguished HAI and CAI, the percentage of *P. aeruginosa* isolates exhibiting resistance against ceftazidime was 27.6% (21.5–34.5; 41/143) and 15.8% (3/19); piperacillin/tazobactam 62.5% (10/16) and 52.6% (10/19); carbapenems 32.4% (28.3–36.8; 143/442) and 36.8% (7/19); fluoroquinolones 37.5% (6/16) and 63.2% (12/19); gentamicin 30.0% (23.8–37.1; 49/166) and 31.6% (6/19); amikacin 25.0% (19.3–31.9; 42/169) and 26.3% (5/19); and tobramycin 46.6% (27/58) and unknown (no CAI data) respectively. Compared to previous years (2015–2019), recent (2020–2023) percentages of *P. aeruginosa* isolates exhibiting resistance were higher for fluoroquinolones (56.2% [45.1–66.7] vs 31.8% [24.3–40.3]); lower for tobramycin (36.5% vs 54.0% [43.6–64.1]); and were stable for ceftazidime (24.1% [18.8–30.4] vs 32.8% [27.7–38.4]), carbapenems (38.8% [29.7–48.8] vs 25.1% [19.8–31.2]), gentamicin (32.3% [26.5–38.6] vs 33.5% [28.5–39.0]), and amikacin (25.6% [20.4–31.6] vs 25.5% [20.8–30.8]), and piperacillin/tazobactam (27.0% [20.3–34.8] vs 28.4% [20.3–38.2]) (Fig. 5).

In communities, the percentage of *P. aeruginosa* isolates exhibiting resistance against carbapenems was 9.0% (3.9–19.2; 3/36); ceftazidime 8.8% (95% CI 3.6–20.2; 4/48); fluoroquinolones 7.8% (3.1–18.1; 5/54); gentamicin 10.8% (5.1–21.5; 6/57); amikacin 1.9% (1/54); and tobramycin 12.3% (7/57).

#### Staphylococcus aureus

28 reports described 3323 *S. aureus* isolates, including 25 hospital reports (2507 isolates) and 6 community reports (816 isolates).

In hospitals, the percentage of *S. aureus* isolates exhibiting resistance against methicillin was 22.2% (95% CI 19.5–25.1; 188/831); vancomycin 5.0% (4.1–6.1; 57/1686); clindamycin 22.0% (18.0–26.5; 63/363); and erythromycin 28.6% (24.5–33.0; 122/427) (Fig. 4, Supplementary Fig. S1 and Table S11). Across the five hospital reports that distinguished HAI and CAI, the percentage of *S. aureus* isolates exhibiting resistance against methicillin was unknown (no HAI data) and 62.5% (5/8); vancomycin 7.2% (4.9–10.5; 24/324) and 0.0% (0/9); clindamycin 17.0% (13.3–21.3; 36/247) and unknown (no CAI data); and erythromycin 40.0% (2/3) and unknown (no CAI data), respectively. Compared to previous years (2015–2019), recent (2020–2023) percentages of *S. aureus* isolates exhibiting resistance was lower for vancomycin (0.7% [0.1–3.7] vs 10.3% [7.3–14.3]); and stable for methicillin (26.3% [11.0–50.8] vs 25.2% [21.7–29.1]) and clindamycin (24.3% [15.9–35.4] vs 14.2% [10.6–18.8]).

In communities, the percentage of *S. aureus* isolates exhibiting resistance against methicillin was 11.1% (95% CI 8.3–14.7; 9/367); vancomycin 2.2% (0.9–5.5; 4/185); clindamycin 9.6% (5.9–15.4%; 10/150); and erythromycin 10.4% (7.5–14.3; 14/317).

#### Acinetobacter baumannii

27 reports described 2832 *A. baumannii* isolates, including 256 hospital reports (2832 isolates) and 2 community reports (57 isolates).

In hospitals, the percentage of *A. baumannii* isolates exhibiting resistance against carbapenems was 70.7% (95% CI 67.4–73.8; 546/769); gentamicin 67.9% (65.9–69.8; 1493/2198); amikacin 31.0% (29.1–32.9; 691/2231); tigecycline 30.4% (28.3–32.5; 546/1803) and colistin 47.5% (37.9–57.2; 47/99) (Fig. 4, Supplementary Fig. S1 and Table S10). Across the five hospital reports that distinguished HAI and CAI, the percentage of *A. baumannii* isolates exhibiting resistance against carbapenems was 93.6% (90.5–95.7; 320/342) and 50.0% (14/28); gentamicin 85.3% (75.6–91.6; 64/75) and 21.4% (6/28); amikacin 49.3% (38.3–60.4; 37/75) and 17.9% (5/28); and tigecycline 46.6% (27/58) and unknown (no CAI data) respectively. Compared to previous years (2012–2017), recent (2018–2023) percentages of *A. baumannii* isolates exhibiting resistance were higher for gentamicin (78.8% [73.2–83.5] vs 66.1% [63.9–68.2]), amikacin (42.9% [36.7–49.3] vs 28.8% [26.8–30.9]), tigecycline (40.0% [29.3–51.8] vs 29.7% [27.5–32.0]), and colistin (97.3% vs 20.4 [11.8–32.9]); and was lower for carbapenems (33.3% vs 53.2% [48.3–58.1]) (Fig. 5).

In communities, the percentage of *A. baumannii* isolates exhibiting resistance against carbapenems was 23.5% (8/34); gentamicin 24.4% (95% CI 14.3–38.4; 11/46); amikacin 27.7% (13/47); and tigecycline 19.1% (9/47).

#### Streptococcus pneumoniae

18 reports described 799 *S. pneumoniae* isolates, including 8 hospital reports (163 isolates) and 10 community reports (636 isolates).

In hospitals, the percentage of *S. pneumoniae* isolates exhibiting resistance against penicillin G was 29.9% (95% CI 21.3–40.2; 26/87); 3GC 16.4% (8.9–28.3; 9/55); co-trimoxazole 39.8% (30.8–49.5; 40/102); oxacillin 34.0% (22.4–47.8; 17/50); erythromycin 21.2% (13.1–32.5; 14/66); and carbapenems 13.5% (5/37) (Fig. 4, Supplementary Fig. S1 and Table S12). Across the three hospital reports that distinguished HAI and CAI, the percentage of *S. pneumoniae* isolates exhibiting resistance against penicillin G was 66.7% (2/3) and 40.0% (22/55); co-trimoxazole 24.3% (9/37) and unknown (no CAI data); 3GC unknown (no HAI data) and 9.8% (4.6–17.8%; 9/92); and carbapenems unknown (no HAI data) and 15.6% (5/32) respectively.

In communities, the percentage of *S. pneumoniae* isolates exhibiting resistance against penicillin G was 23.9% (34/142); co-trimoxazole 50.6% (46.7–54.4; 320/633); oxacillin 45.5% (40.5–50.6; 167/367) and erythromycin 15.5% (12.6–18.9; 80/516).

#### *Salmonella enterica* serovar Typhi and Paratyphi A (typhoidal)

7 reports described 562 *S. enterica serovar* Typhi and Paratyphi A (typhoidal) isolates, including 6 hospital reports (300 isolates) and 1 community report (262 isolates).

In hospitals, the percentage of *S. enterica serovar* Typhi and Paratyphi A (typhoidal) isolates exhibiting resistance against fluoroquinolones was 2.8% (95% CI 1.4–5.4; 8/287); co-trimoxazole 12.4% (8.4–17.9; 23/186); 3GC 4.7% (2.6–8.2; 11/230); ampicillin 7.0% (4.2–11.7; 13/185); and chloramphenicol 17.6% (3/17) (Fig. 4, and Supplementary Fig. S1 and Table S13).

In communities, the percentage of *S. enterica serovar* Typhi and Paratyphi A (typhoidal) isolates exhibiting resistance against fluoroquinolones, 3GC and co-trimoxazole was 0.0% (each 0/262), and against ampicillin and chloramphenicol was 0.8% (both 2/262).

#### Neisseria gonorrhoeae

6 reports described 629 *N. gonorrhoeae* isolates, all in communities. The percentage of *N. gonorrhoeae* isolates exhibiting resistance against fluoroquinolones was 58.4% (95% CI 54.4–62.3; 347/594); 3GC 3.5% (2.3–5.2; 22/629); penicillin G 98.2% (160/163), azithromycin 1.1% (0.5–2.3; 6/568); and spectinomycin 0.2% (0.0–1.1; 1/489) (Fig. 4, Supplementary Fig. S1 and Table S14).

#### *Shigella* spp.

5 reports described 1164 *Shigella spp*. isolates, including 4 hospital reports (1107 isolates) and 1 community report (57 isolates). All reports were published more than 5 years ago.

In hospitals, the percentage of *Shigella spp*. isolates exhibiting resistance against fluoroquinolones was 0.4% (95% CI 0.1–0.9; 4/1107); 3GC 5.1% (2/39); and co-trimoxazole 68.5% (65.7–71.3; 732/1068) (Fig. 4, Supplementary Fig. S1 and Table S15).

In communities, the percentage of *Shigella spp*. isolates exhibiting resistance against fluoroquinolones and 3GC was 0.0% (both 0/57); co-trimoxazole 70.2% (40/57); and azithromycin 3.5% (2/57).

#### Haemophilus influenzae

3 reports described 192 *H. influenzae* isolates, including 2 hospital reports (107 isolates) and 1 community report (85 isolates).

In hospitals, the percentage of *H. influenzae* isolates exhibiting resistance against ampicillin was 38.2% (13/34); 3GC 2.9% (1/34); fluoroquinolones 3.7% (95% CI 1.5–9.2; 4/107); and co-trimoxazole 58.8% (20/34) (Fig. 4, Supplementary Fig. S1 and Table S16).

In communities, the percentage of *H. influenzae* isolates exhibiting resistance against ampicillin was 14.1% (12/85); 3GC and fluoroquinolones 0.0% (both 0/85); co-trimoxazole 25.9% (22/85); and amoxicillin-clavulanic acid 1.2% (1/85).

#### *Salmonella spp*. (non-typhoidal)

3 reports described 259 *Salmonella spp* (non-typhoidal) isolates, including 2 reports (176 isolates) in hospitals and 2 reports (83 isolates) in communities.

In hospitals, the percentage of *Salmonella spp* (non-typhoidal) isolates exhibiting resistance against fluoroquinolones was 16.7% (3/18); 3GC 26.7% (4/15); carbapenems 16.7% (2/12); and co-trimoxazole 8.2% (13/158) (Fig. 4, and Supplementary Fig. S1 and Table S17).

In communities, the percentage of *Salmonella spp* (non-typhoidal) isolates exhibiting resistance against fluoroquinolones was 71.1% (95% CI 60.6–79.7; 59/83); 3GC 70.6% (59.6–79.6; 58/77); carbapenems 5.3% (1/19); and co-trimoxazole 8.9% (5/56).

#### Neisseria meningitidis

None of the included reports reported resistance data for *N. meningitidis*.

## Discussion

The best represented GLASS target bacterial pathogens in this systematic review were *E. coli, K. pneumoniae, P. aeruginosa, S. aureus, A. baumannii,* and *S. pneumoniae*, whereas data were limited for *Shigella* spp., typhoidal and non-typhoidal *Salmonella*, *N. gonorrhoeae*, and *H. influenzae*, and there were no reports included for *N. meningitidis*. Although the differential representation of the GLASS target pathogens may partly reflect differences in their disease burdens, the data likely overrepresented hospital settings (72% of studies), given that microbiology laboratories and clinical services in Indonesia often prioritise taking bacterial cultures in hospitalised patients, over testing infections in community settings (24% of studies), such as for diarrhoeal illness and genito-urinary infections.^15^^,^^16^ The evidence base was found to be uneven with around 76% of studies from Java, leaving other geographic areas underrepresented. Gaps in classification metadata (e.g. CAI/HAI, hospitalisation status) limited the ability to draw firm conclusions. Nearly half of the publications appeared during the most recent five years, reflecting the growing importance of AMR on the national and global health agenda. There is an urgent need to include the GLASS target pathogens for which existing data were limited for Indonesia in future AMR surveillance and research. This is particularly true for *Shigella spp*., accounting for 13% of diarrhoeal deaths globally,^17^ and *N. gonorrhoeae*, causing substantial morbidity in LMICs,^18^ and which are both known for rapid development of AMR.

The overall AMR prevalence estimates were higher in hospitals, compared to community settings, as expected, most likely due to more and broader-spectrum antibiotic use (hence selection pressure) among vulnerable patients, coupled with poor infection and prevention control. AMR estimates for common bacterial pathogens to many of the most accessible and widely used antibiotics in Indonesian hospitalised populations were among the highest reported for the Southeast Asia region,^8^^,^^19^, ^20^, ^21^ and higher than reported for the European Union.^22^ These findings largely corroborate previous reports on culture data from routine clinical practice in sentinel Indonesian hospitals in 2022, which were not included in this review (Panel).^8^^,^^10^ This review found that in hospital settings, around 22% of *K. pneumoniae*, 18% of *E. coli*, 36% of *P. aeruginosa* and 71% of *A. baumannii* isolates were carbapenem-resistant, all classified as Priority 1 (“critical”) on the WHO priority pathogen list for research and development of new antibiotics.^23^ Moreover, the rising resistance levels to carbapenems in *K. pneumoniae* and *E. coli* over time means that the management of severe infections associated with Enterobacterales is increasingly dependent on more expensive and less readily available antibiotics. Concerningly high levels of carbapenem-resistant *A. baumannii* have been reported across Southeast Asia,^8^^,^^19^, ^20^, ^21^ posing an emerging threat to hospitalised populations globally.^24^^,^^25^ Across the subset of studies that reported on resistance mechanisms, the proportion of ESBL-producing isolates was 60.1% for *E. coli* (95% CI 56.3–63.7; 6 reports), and 67.4% for *K. pneumoniae* (95% CI 63.7–71.0; 8 reports), which was in the same range as the overall prevalence among included hospital studies of 3GC-resistance (considered a proxy for ESBL), at 66.4% (95% CI 64.0–68.6; 31 reports) for *E. coli* and 74.4% (95% CI 72.3–76.4; 27 reports) for *K. pneumoniae*. In a study of *E. coli* and *K. pneumoniae* isolates in Surabaya, *bla*_CTX-M-15_ was the predominant ESBL-gene.^26^ With regards to carbapenemases, studies in intensive care patients in Jakarta have identified the *bla*_NDM_ gene as most frequent in *K. pneumoniae* isolates,^27^ *bla*_VIM_, *bla*_IMP_ and *bla*_GES-5_ gene in *P. aeruginosa* isolates,^28^ and *bla*_OXA-23_-like gene in *A. baumannii*.^29^

In Indonesian hospital populations, penicillin-resistant *S. pneumoniae* (30%) was higher than reported in the Philippines^21^ and Malaysia,^19^ but lower than in Thailand^8^ and Vietnam^20^; and methicillin-resistant *S. aureus* (22%) was lower than in previous reports (38–40%),^8^^,^^10^ as well as in most other countries in the region (Panel).^8^^,^^20^^,^^21^ With regard to community-based studies, available data suggested considerable AMR levels (e.g. carbapenem-resistant *E. coli* and *K. pneumoniae* at 16% and 28%, respectively; penicillin-resistant *S. pneumoniae* at 24%; and methicillin-resistant *S. aureus* at 11%), although the limited community-level evidence base call for more granular, representative surveys to inform public health policy. Moreover, comparisons of AMR prevalence across time periods were only feasible for *K. pneumoniae, E. coli, A. baumannii* and *P. aeruginosa* in hospital settings, due to limited available data for the other pathogens.

A range of complex drivers render Indonesia particularly vulnerable to antibiotic-resistant bacteria. The widespread and weakly regulated use of antimicrobial agents in human and veterinary medicine and aquaculture for therapeutic or prophylactic purposes is the main driver of the acquisition and selection of antibiotic-resistant bacteria.^1^^,^^3^ Based on pharmaceutical sales data, Indonesia has seen an estimated 2.5-fold increase in nationwide antibiotic consumption between 2000 and 2015, mostly broad-spectrum penicillins, fluoroquinolones and cephalosporins, placing Indonesia among the greatest risers in antibiotic consumption globally (ranked 29th of 76 countries analysed).^30^ A recent literature review found the appropriateness of antibiotic prescribing to be low, coupled with widespread over-the-counter use of non-prescription antibiotics.^31^ In many underdeveloped, both urban and rural, settings across Indonesia, AMR emergence and spread is likely exacerbated by inadequate sanitation and hygiene, poor infection prevention and control in health care facilities, and lack of awareness of AMR in communities and among healthcare providers.^31^^,^^32^

The implementation of Indonesia’s 2020–2024 National Action Plan on AMR has shown progress in strengthening national capacities for microbiological laboratories and surveillance,^33^ renewed national guidelines for antibiotic prescribing^34^ and recent regulations for hospital antimicrobial stewardship programmes.^35^ Nonetheless, to build sustainable capacity to contain AMR, a recent analysis identified several urgent policy priorities, that include implementing nationwide surveillance of AMR and antimicrobial consumption and use; evidence-based antimicrobial stewardship and infection prevention and control programmes; developing regulatory frameworks to control antimicrobial use; amongst others.^36^ Further investments will be especially needed to strengthen the quality of primary health care delivery, diagnostic laboratory capacities, and health information systems, with particular attention to equitable access to timely and reliable infection diagnostics and appropriate antibiotic prescribing across all healthcare settings.

There are several limitations to this review. First, the substantial variability in populations, methodology and laboratory methods between studies limited the ability for data aggregation. Although most studies were based on routinely collected clinical specimens, the AMR data included in this systematic review were not derived from representative sentinel surveillance sites, as recommended by GLASS, which may have introduced several potential biases that may have led to over- or underestimation of AMR prevalence. Because we had to rely on aggregated, author-reported AMR data for most studies, we could not ascertain the clinical significance of bacterial isolates and we may thus have included non-pathogenic colonizing pathogens, for which antimicrobial susceptibilities may differ. GLASS acknowledges this risk as an inherent limitation to their approach.^12^ Nonetheless, the main AMR estimates in the systematic review were in the same range as those reported in previous reports (not included in this review), as summarised in the Panel**,** suggesting that any potential bias from including colonizing pathogens may have been limited. Second, in the Indonesian context, microbiology laboratory capability has been underdeveloped and culture utilization has been very low.^15^^,^^16^ Therefore, the peer-reviewed literature on AMR epidemiology may overrepresent hospitals that have microbiological laboratory capacity, as well as complex patient populations with high antibiotic exposure and who therefore have a higher risk of a drug-resistant infection. Third, the absence of data on laboratory quality assurance, standardised test panels of antibiotics, the lack of reported minimal inhibitory concentrations and the use of different CLSI/EUCAST versions during the study period (2000–2023) may have introduced heterogeneity in AST interpretation. This issue has been previously recognised and we therefore applied the MICRO checklist to ensure that we reported the microbiological data transparently.^13^ Although guidelines for clinical breakpoints were not available for some pathogen-antimicrobial combinations (e.g. fosfomycin for *K. pneumoniae*), we opted to include them for the purpose of this epidemiological evidence synthesis. Lastly, although our comprehensive search strategy included the main national and global bibliographic databases, we cannot rule out that we may have missed other relevant publications. Furthermore, the exclusion of non-peer-reviewed, grey literature meant that we might not have included useful evidence from other sources.^8^^,^^10^ However, their exclusion likely improved the quality of the evidence as grey literature may not always follow recommended quality standards for evaluation.

In conclusion, AMR prevalence estimates in common and medically important gram-negative bacteria in Indonesia are among the highest reported in the Southeast Asian region. More representative, granular, high-quality and standardised AMR data are required to construct accurate AMR estimates for all GLASS-specific pathogens, geographic areas, across the national, province, district and health facility levels. This information can inform locally relevant empiric treatment guidelines and effective public health policies and interventions, and guide priorities for the National Action Plan on AMR.

## Contributors

MG and RLH conceptualised the study. MG, DMS, RL, HRVD and RLH designed the study protocol and data extraction instrument. MG, GL, DMS and RS collected and verified the data, overseen by RL, HRVD and RLH. MG, GL and RLH performed the data analysis and had full access to all study data. MG, GL, RS, and RLH drafted the paper, with critical inputs from YRS, RL, EJN, HRVD, and AK. All authors had full access to all the data in the study, critically revised the manuscript, accept responsibility to submit for publication, and gave approval for the final version to be published.

## Data sharing statement

No additional data are available.

## Editor note

The Lancet Group takes a neutral position with respect to territorial claims in published maps and institutional affiliations.

## Declaration of interests

HRVD serves as Board Member of The Wellcome Surveillance and Epidemiology of Drug-resistant Infections Consortium (SEDRIC). AK serves as the current Chair of the National AMR Committee (Komite Pengendalian Resistensi Antimikroba). The other authors declare no competing interests.

## Figures and Tables

**Supplementary Fig. S1:**