# Supplementary material for: Antimicrobial resistance among common bacterial pathogens in Indonesia: a systematic review
Source: Lancet Reg Health Southeast Asia. 2024 May 13;26:100414. doi: 10.1016/j.lansea.2024.100414 (PMC11109028; doi:10.1016/j.lansea.2024.100414)
Supplement: Supplementary Tables S1–S17 [file mmc1.docx]

**SUPPLEMENTARY MATERIAL**

**Antibiotic resistance among common bacterial pathogens in Indonesia: a systematic review**

|  | **Page** |
| --- | --- |
| **Table S1.** Search strategy (up to 25 May 2023) | 2 |
| **Table S2.** Checklist of selected items from the Strengthening the Reporting of Observational Studies in Epidemiology (STROBE) statement | 3 |
| **Table S3.** MICRO checklist of 13 mandatory items for reporting human clinical microbiological data in the 99 included studies | 4 |
| **Table S4.** Pathogen-antimicrobial combinations included in this review | 10 |
| **Table S5.** Summary of individual reports included in the review | 12 |
| **Table S6.** Summary of number of bacterial isolates and specimen types included in the review for each GLASS pathogen | 20 |
| **Figure S1.** AMR prevalence estimates for all GLASS-specific pathogen-antimicrobial combinations in hospital and community settings. | 21 |
| **Table S7.** Numbers and proportions of resistant isolates for *Klebsiella pneumoniae*-antimicrobial combinations | 23 |
| **Table S8.** Numbers and proportions of resistant isolates for *Escherichia coli*-antimicrobial combinations | 25 |
| **Table S9.** Numbers and proportions of resistant isolates for *Pseudomonas aeruginosa*-antimicrobial combinations | 27 |
| **Table S10.** Numbers and proportions of resistant isolates for *Staphylococcus aureus*-antimicrobial combinations | 28 |
| **Table S11.** Numbers and proportions of resistant isolates for *Acinetobacter baumannii*-antimicrobial combinations | 29 |
| **Table S12.** Numbers and proportions of resistant isolates for *Streptococcus pneumoniae*-antimicrobial combinations | 30 |
| **Table S13.** Numbers and proportions of resistant isolates for *Salmonella enterica* serovar Typhi and Paratyphi A (typhoidal)-antimicrobial combinations | 31 |
| **Table S14.** Numbers and proportions of resistant isolates for *Neisseria gonorrhoeae*-antimicrobial combinations | 32 |
| **Table S15.** Numbers and proportions of resistant isolates for *Shigella spp*.-antimicrobial combinations | 33 |
| **Table S16.** Numbers and proportions of resistant isolates for *Haemophilus influenzae*-antimicrobial combinations | 33 |
| **Table S17.** Numbers and proportions of resistant isolates for *Salmonella spp.* (non-typhoidal)-antimicrobial combinations | 34 |
| References | 35 |

# Table S1. Search strategy (up to 25 May 2023)

| Database | Search keywords | Hits |
| --- | --- | --- |
| PubMed | (("indonesia"[MeSH Terms] OR "indonesia"[All Fields] OR "indonesia s"[All Fields] OR "indonesias"[All Fields]) AND ("drug resistance"[MeSH Terms] OR "antimicrobial resistance"[All Fields] OR "antibiotic resistance"[All Fields] OR "susceptibility"[All Fields] OR "resistance"[All Fields]) AND ("Escherichia coli"[MeSH Terms] OR "Klebsiella"[MeSH Terms] OR "Acinetobacter"[MeSH Terms] OR "Staphylococcus aureus"[MeSH Terms] OR "Streptococcus pneumoniae"[MeSH Terms] OR "Salmonella"[MeSH Terms] OR "Shigella"[MeSH Terms] OR "Neisseria gonorrhoeae"[MeSH Terms] OR "Pseudomonas aeruginosa"[MeSH Terms] OR "Neisseria meningitidis"[MeSH Terms] OR "Haemophilus influenzae"[MeSH Terms] OR "MRSA"[All Fields] OR "methicillin-resistant Staphylococcus aureus"[All Fields] OR "ESBL"[All Fields] OR "extended spectrum beta-lactamases"[All Fields])) AND (2000:2023[pdat]) | 205 |
| Embase | 1. 'Indonesia'/exp 2. 'drug resistance'/exp 3. “antimicrobial resistance” OR “antibiotic resistance” OR “susceptibility” OR “resistance” 4. #2 OR #3 5. 'escherichia coli'/exp OR 'escherichia coli' OR 'klebsiella'/exp OR 'klebsiella' OR 'acinetobacter'/exp OR 'acinetobacter' OR 'neisseria gonorrhoeae'/exp OR 'neisseria gonorrhoeae' OR 'pseudomonas aeruginosa'/exp OR 'pseudomonas aeruginosa' OR 'salmonella'/exp OR 'salmonella' OR 'shigella'/exp OR 'shigella' OR 'staphylococcus aureus'/exp OR 'staphylococcus aureus' OR 'streptococcus pneumoniae'/exp OR 'streptococcus pneumoniae' OR 'neisseria meningitidis'/exp OR 'neisseria meningitidis' OR 'haemophilus influenzae'/exp OR 'haemophilus influenzae' 6. “MRSA” OR “methicillin-resistant Staphylococcus aureus” OR “ESBL” OR “extended spectrum beta-lactamases” 7. #5 OR #6 8. #1 AND #4 AND #7 9. #8 AND (2000:py OR 2001:py OR 2002:py OR 2003:py OR 2004:py OR 2005:py OR 2006:py OR 2007:py OR 2008:py OR 2009:py OR 2010:py OR 2011:py OR 2012:py OR 2013:py OR 2014:py OR 2015:py OR 2016:py OR 2017:py OR 2018:py OR 2019:py OR 2020:py OR 2021:py OR 2022:py OR 2023:py) | 719 |
| Garba Rujukan Digital (GARUDA) | 1. antibiotic resistance, by title , from: 2000 , to: 2023 [66 hits] 2. antimicrobial resistance, by title , from: 2000 , to: 2023 [42 hits] 3. resistensi antibiotik, by title , from: 2000 , to: 2023 [119 hits] 4. resistensi antimikroba, by title , from: 2000 , to: 2023 [13 hits] | 240 |
| Neliti | 1. “antibiotic resistance” (filter: tanggal: 2000-2023) [133 hits] 2. “antimicrobial resistance” (filter: tanggal: 2000-2023) [133 hits] 3. “resistensi antibiotik” (filter: tanggal: 2000-2023) [120 hits] 4. “resistensi antimikroba” (filter: tanggal: 2000-2023) [380 hits] | 647 |
| Jurnal Penelitian dan Pengembangan Pelayanan Kesehatan | 1. antibiotic resistance [6 hits] 2. antimicrobial resistance [2 hit] 3. resistensi antibiotik [7 hits] 4. resistensi antimikroba [3 hit] | 18 |

# Table S2. Checklist of selected items from the STrengthening the Reporting of Observational Studies in Epidemiology (STROBE) statement

| Section | | Item No | Recommendation |
| --- | --- | --- | --- |
| Methods | Study design | 4 | Present key elements of study design |
|  | Setting | 5 | Describe study’s setting (hospital or community), geographic location, and period of data collection |
| Results | Descriptive data | 14 | Denominators and numerators of susceptible or non-susceptible samples (or can be calculated from data presented) |
|  | Outcome data | 15 | *Cohort study*—Report numbers of outcome events or summary measures over time  *Case-control study*—Report numbers in each exposure category, or summary measures of exposure  *Cross-sectional study*—Report numbers of outcome events or summary measures |

# Table S3. MICRO checklist of 13 mandatory items for reporting human clinical microbiological data in the 102 included studies

| First author,  Year of publication | **1.Specimen type** | **2.Sampling period** | **3.Sampling strategy** | **5.Geographical setting** | **6.Clinical setting** | **8.Target organism identification** | **9.Antimicrobial susceptibility testing** | **11.AMR definitions** | **12.External QA** | **14.Duplicate and sequential isolates** | **15.Population** | **16.Denominators** | **18.Resistance proportions for single agent/class** | **Number of items missing in each study** |
| --- | --- | --- | --- | --- | --- | --- | --- | --- | --- | --- | --- | --- | --- | --- |
| Adhima, 2022^1^ | ● | ● | ● | ● | ● | ● | ● | ○ | ○ | ○ | ● | ● | ● | 3 |
| Afifurrahman, 2014^2^ | ● | ● | ● | ● | ● | ● | ○ | ○ | ○ | ○ | ○ | ● | ● | 5 |
| Akbar, 2014^3^ | ● | ● | ● | ● | ● | ● | ○ | ○ | ○ | ○ | ● | ● | ● | 4 |
| Amanda, 2020^4^ | ● | ● | ● | ● | ● | ● | ● | ● | ○ | ○ | ● | ● | ● | 2 |
| Anggraini, 2022^5^ | ● | ● | ● | ● | ● | ● | ● | ● | ○ | ○ | ○ | ● | ● | 3 |
| Ariana, 2020^6^ | ○ | ● | ● | ● | ● | ○ | ○ | ○ | ○ | ○ | ○ | ● | ● | 7 |
| Artono, 2022^7^ | ● | ● | ● | ● | ● | ● | ● | ○ | ○ | ● | ● | ● | ● | 2 |
| Azwar, 2012^8^ | ● | ● | ● | ● | ● | ○ | ○ | ○ | ○ | ○ | ● | ● | ● | 5 |
| Bukitwetan, 2007^9^ | ● | ● | ● | ● | ● | ● | ● | ○ | ○ | ○ | ● | ● | ● | 3 |
| Chudlori, 2012^10^ | ● | ● | ● | ● | ● | ● | ● | ○ | ○ | ○ | ● | ● | ● | 3 |
| Chung, 2011^11^ | ● | ● | ● | ● | ● | ● | ● | ● | ○ | ○ | ● | ● | ● | 2 |
| Deurenberg, 2009^12^ | ● | ● | ● | ● | ● | ● | ● | ○ | ○ | ○ | ○ | ● | ● | 4 |
| Devian, 2022^13^ | ● | ● | ● | ● | ● | ● | ○ | ○ | ○ | ○ | ● | ● | ● | 4 |
| Donegan, 2006^14^ | ● | ● | ● | ● | ● | ● | ● | ● | ○ | ○ | ● | ● | ● | 2 |
| Dwipoerwantoro, 2005^15^ | ● | ● | ● | ● | ● | ● | ○ | ○ | ○ | ○ | ● | ● | ● | 4 |
| Fabian, 2020^16^ | ○ | ● | ○ | ● | ○ | ○ | ○ | ● | ○ | ○ | ○ | ● | ● | 8 |
| Farida, 2013^17^ | ● | ● | ● | ● | ● | ● | ● | ○ | ○ | ○ | ● | ● | ● | 3 |
| Farida, 2014^18^ | ● | ● | ● | ● | ● | ● | ● | ○ | ○ | ○ | ● | ● | ● | 3 |
| Fathin, 2022^19^ | ● | ● | ● | ● | ● | ● | ● | ○ | ○ | ○ | ● | ● | ● | 3 |
| Firdiana, 2016^20^ | ● | ● | ● | ● | ○ | ○ | ● | ● | ○ | ○ | ● | ● | ● | 4 |
| Hananta, 2016^21^ | ● | ● | ● | ● | ● | ● | ● | ● | ● | ○ | ● | ● | ● | 1 |
| Harimurti, 2016^22^ | ● | ● | ● | ● | ● | ● | ● | ○ | ○ | ○ | ● | ● | ● | 3 |
| Harimurti, 2021^23^ | ● | ● | ● | ● | ● | ● | ● | ● | ○ | ● | ● | ● | ● | 1 |
| Harnanik, 2005^24^ | ● | ● | ○ | ● | ● | ● | ● | ○ | ○ | ○ | ○ | ● | ● | 5 |
| Herdiyanti, 2019^25^ | ● | ● | ● | ● | ● | ○ | ○ | ○ | ○ | ○ | ● | ● | ● | 5 |
| Herwana, 2010^26^ | ● | ● | ● | ● | ● | ● | ● | ○ | ○ | ○ | ● | ● | ● | 3 |
| Hidayah, 2020^27^ | ● | ● | ● | ● | ● | ● | ○ | ○ | ○ | ● | ● | ● | ● | 3 |
| Indraningrat, 2022^28^ | ● | ● | ● | ● | ● | ● | ● | ○ | ○ | ○ | ● | ● | ● | 3 |
| Indri, 2015^29^ | ● | ● | ● | ● | ● | ○ | ○ | ○ | ○ | ○ | ● | ● | ● | 5 |
| Indriatmi, 2019^30^ | ● | ● | ● | ● | ● | ● | ● | ● | ○ | ○ | ● | ● | ● | 2 |
| Karmila, 2022^31^ | ● | ● | ● | ● | ● | ● | ● | ● | ○ | ○ | ● | ● | ● | 2 |
| Kartasasmita, 2002^32^ | ● | ● | ● | ● | ● | ● | ● | ○ | ○ | ○ | ● | ● | ● | 3 |
| Khalilullah, 2011^33^ | ● | ● | ● | ● | ● | ● | ● | ○ | ○ | ○ | ○ | ● | ● | 3 |
| Kitagawa, 2018^34^ | ● | ● | ● | ● | ● | ● | ● | ○ | ○ | ○ | ● | ● | ● | 4 |
| Lestari, 2008^35^ | ● | ● | ● | ● | ● | ● | ● | ○ | ○ | ○ | ○ | ● | ● | 4 |
| Lestari, 2010^36^ | ● | ● | ● | ● | ● | ● | ● | ● | ○ | ○ | ● | ● | ● | 2 |
| Lestari, 2013^37^ | ● | ● | ● | ● | ● | ○ | ○ | ○ | ○ | ○ | ○ | ● | ● | 6 |
| Lugito, 2017^38^ | ● | ● | ● | ● | ● | ● | ● | ○ | ○ | ○ | ● | ● | ● | 3 |
| Mayasari, 2017^39^ | ● | ● | ○ | ● | ● | ● | ○ | ○ | ○ | ○ | ● | ● | ● | 5 |
| Meriyani, 2021^40^ | ● | ● | ● | ● | ● | ● | ● | ○ | ○ | ○ | ● | ● | ● | 3 |
| Meta, 2014^41^ | ● | ● | ● | ● | ● | ● | ○ | ○ | ○ | ○ | ● | ● | ● | 4 |
| Moehario, 2009^42^ | ● | ● | ● | ● | ● | ○ | ● | ○ | ○ | ○ | ● | ● | ● | 4 |
| Moehario, 2020^43^ | ● | ● | ● | ● | ● | ● | ● | ○ | ○ | ○ | ● | ● | ● | 3 |
| Muktiarti, 2021^44^ | ● | ● | ● | ● | ○ | ● | ● | ● | ○ | ● | ○ | ● | ● | 3 |
| Murni, 2016^45^ | ● | ● | ● | ● | ● | ● | ● | ● | ○ | ● | ● | ● | ● | 1 |
| Nathania, 2022^46^ | ● | ● | ○ | ● | ○ | ● | ● | ○ | ○ | ○ | ○ | ● | ● | 6 |
| Nirwati, 2018^47^ | ● | ● | ○ | ● | ● | ● | ● | ○ | ○ | ○ | ● | ● | ● | 4 |
| Nirwati, 2019^48^ | ● | ● | ○ | ● | ● | ● | ● | ● | ○ | ○ | ● | ● | ● | 3 |
| Nur, 2016^49^ | ● | ● | ● | ● | ● | ● | ● | ● | ○ | ○ | ● | ● | ● | 2 |
| Oyofo, 2002^50^ | ● | ● | ● | ● | ● | ● | ○ | ○ | ○ | ○ | ● | ● | ● | 4 |
| Parut, 2015^51^ | ● | ● | ● | ● | ● | ● | ● | ● | ○ | ○ | ● | ● | ● | 2 |
| Patilaya, 2019^52^ | ● | ● | ○ | ● | ○ | ● | ● | ● | ○ | ○ | ○ | ● | ● | 5 |
| Pelegrin, 2019^53^ | ● | ● | ● | ● | ● | ● | ● | ○ | ○ | ○ | ○ | ● | ● | 4 |
| Permana, 2022^54^ | ● | ● | ● | ● | ● | ● | ● | ○ | ○ | ● | ● | ● | ● | 2 |
| Prabowo, 2012^55^ | ● | ● | ○ | ● | ● | ● | ● | ○ | ○ | ○ | ● | ● | ● | 4 |
| Punjabi, 2013^56^ | ● | ● | ● | ● | ● | ● | ● | ○ | ● | ● | ● | ● | ● | 1 |
| Purba, 2019^57^ | ● | ● | ● | ● | ● | ○ | ○ | ● | ○ | ○ | ● | ● | ● | 4 |
| Puspandari, 2016^58^ | ● | ○ | ● | ● | ● | ● | ● | ● | ○ | ○ | ● | ● | ● | 3 |
| Rachman, 2016^59^ | ● | ● | ● | ● | ● | ○ | ○ | ○ | ○ | ○ | ○ | ● | ● | 6 |
| Radji, 2011^60^ | ● | ● | ● | ● | ● | ○ | ● | ○ | ○ | ○ | ○ | ● | ● | 5 |
| Radji, 2014^61^ | ● | ● | ● | ● | ● | ● | ● | ○ | ○ | ○ | ● | ● | ● | 3 |
| Rahman, 2015^62^ | ● | ● | ● | ● | ● | ● | ○ | ○ | ○ | ○ | ○ | ● | ● | 5 |
| Ramdhani, 2022^63^ | ○ | ● | ○ | ● | ● | ● | ● | ● | ○ | ○ | ○ | ● | ● | 5 |
| Rosana, 2020a^64^ | ● | ● | ● | ● | ● | ● | ● | ○ | ○ | ● | ● | ● | ● | 2 |
| Rosana, 2020b^65^ | ● | ● | ● | ● | ● | ● | ● | ○ | ○ | ○ | ○ | ● | ● | 4 |
| Rosyid, 2021^66^ | ● | ● | ● | ● | ● | ○ | ● | ● | ○ | ● | ● | ● | ● | 2 |
| Safari, 2014^67^ | ● | ● | ● | ● | ● | ● | ● | ○ | ○ | ○ | ● | ● | ● | 3 |
| Safari, 2015^68^ | ● | ● | ● | ● | ● | ● | ● | ○ | ○ | ○ | ● | ● | ● | 3 |
| Safari, 2020^69^ | ● | ● | ● | ● | ○ | ● | ● | ○ | ○ | ○ | ● | ● | ● | 4 |
| Safari, 2022a^70^ | ● | ● | ● | ● | ● | ● | ● | ○ | ○ | ○ | ○ | ● | ● | 4 |
| Safari, 2022b^71^ | ● | ● | ● | ● | ● | ● | ● | ○ | ○ | ○ | ● | ● | ● | 3 |
| Sahara, 2023^72^ | ● | ● | ● | ● | ● | ● | ● | ○ | ○ | ○ | ○ | ● | ● | 4 |
| Saharman, 2018^73^ | ● | ● | ● | ● | ● | ● | ● | ○ | ○ | ○ | ● | ● | ● | 3 |
| Saharman, 2019^74^ | ● | ● | ● | ● | ● | ● | ● | ○ | ○ | ○ | ● | ● | ● | 3 |
| Saharman, 2020^75^ | ● | ● | ● | ● | ● | ● | ● | ○ | ○ | ○ | ● | ● | ● | 3 |
| Said, 2017^76^ | ● | ● | ● | ● | ● | ● | ● | ● | ○ | ○ | ○ | ● | ● | 3 |
| Salsabila, 2021^77^ | ● | ● | ○ | ● | ● | ● | ● | ● | ○ | ● | ● | ● | ● | 2 |
| Santoso, 2022^78^ | ● | ● | ● | ● | ● | ● | ● | ● | ○ | ● | ● | ● | ● | 1 |
| Saputro, 2013^79^ | ● | ● | ● | ● | ● | ● | ● | ○ | ○ | ○ | ● | ● | ● | 3 |
| Sari, 2015^80^ | ● | ● | ○ | ● | ● | ○ | ○ | ○ | ○ | ○ | ● | ● | ● | 6 |
| Sari, 2017^81^ | ● | ● | ● | ● | ● | ○ | ○ | ○ | ○ | ○ | ● | ● | ● | 5 |
| Setyati, 2012^82^ | ● | ● | ● | ● | ● | ● | ● | ○ | ○ | ○ | ○ | ● | ● | 4 |
| Sholeh, 2020^83^ | ● | ● | ● | ● | ● | ● | ● | ○ | ○ | ● | ● | ● | ● | 2 |
| Sinto, 2022^84^ | ● | ● | ● | ● | ● | ● | ● | ● | ● | ● | ● | ● | ● | 0 |
| Soedarmono, 2022^85^ | ● | ● | ● | ● | ● | ● | ● | ○ | ○ | ○ | ● | ● | ● | 3 |
| Subagdja, 2022^86^ | ○ | ● | ● | ● | ● | ● | ● | ○ | ○ | ○ | ● | ● | ● | 4 |
| Sugianli, 2017^87^ | ● | ● | ● | ● | ● | ● | ● | ● | ● | ○ | ● | ● | ● | 1 |
| Suranadi, 2021^88^ | ● | ● | ● | ● | ● | ● | ● | ○ | ○ | ● | ● | ● | ● | 2 |
| Sutrisna, 2006^91^ | ● | ● | ● | ● | ● | ● | ● | ○ | ○ | ○ | ○ | ● | ● | 4 |
| Tan, 2021^92^ | ● | ● | ● | ● | ● | ● | ● | ○ | ○ | ● | ● | ● | ● | 2 |
| Tandiono, 2022^93^ | ● | ● | ● | ● | ● | ○ | ● | ● | ○ | ○ | ● | ● | ● | 3 |
| Tauran, 2022^94^ | ● | ● | ● | ● | ● | ● | ● | ● | ○ | ● | ● | ● | ● | 1 |
| Tjaniadi, 2003^96^ | ● | ● | ● | ● | ● | ● | ● | ● | ○ | ● | ● | ● | ● | 1 |
| Tjoa, 2013^97^ | ● | ● | ● | ● | ● | ● | ● | ○ | ○ | ○ | ○ | ● | ● | 4 |
| Wang, 2011^98^ | ● | ● | ● | ● | ● | ○ | ● | ● | ○ | ○ | ○ | ● | ● | 4 |
| Wardhana, 2017^99^ | ● | ● | ● | ● | ● | ○ | ○ | ○ | ○ | ○ | ● | ● | ● | 5 |
| Wijaksana, 2019^100^ | ● | ● | ● | ● | ● | ○ | ○ | ○ | ○ | ○ | ● | ● | ● | 5 |
| Wikaningtyas, 2015^101^ | ● | ● | ● | ● | ● | ○ | ● | ○ | ○ | ○ | ○ | ● | ● | 5 |
| Yanagi, 2009^102^ | ● | ● | ● | ● | ● | ● | ● | ● | ○ | ○ | ○ | ● | ● | 3 |
| Number of studies with item missing | 4 | 1 | 11 | 0 | 6 | 19 | 21 | 68 | 95 | 82 | 27 | 0 | 0 | - |
| % of studies with item missing | 4.0% | 1.0% | 11.1% | 0.0% | 6.1% | 19.2% | 21.2% | 68.7% | 96.0% | 82.8% | 27.3% | 0.0% | 0.0% | - |

MICRO checklist item reported (●) or missing (○) from the study

Abbreviations: AMR, antimicrobial resistance; MICRO, Microbiology Investigation Criteria for Reporting Objectively; QA, quality assurance.

# Table S4. Pathogen-antimicrobial combinations included in this review

| Antibiotics | | *A. baumannii* | *E. coli* | *H. influenzae* | *K. pneumoniae* | *N. gonorrhoeae* | *N. meningitidis* | *P. aeruginosa* | *Salmonella spp.* | *Salmonella enterica* serovar Typhi and Paratyphi A | *Shigella spp.* | *S. aureus* | *S. pneumoniae* |
| --- | --- | --- | --- | --- | --- | --- | --- | --- | --- | --- | --- | --- | --- |
| Aminoglycosides | Amikacin | ●○ | ●○ |  | ●○ |  |  | ●○ |  |  |  |  |  |
|  | Gentamicin | ●○ | ●○ |  | ●○ | ●○ |  | ●○ |  |  |  |  |  |
|  | Tobramycin |  |  |  |  |  |  | ●○ |  |  |  |  |  |
| Carbapenems | Doripenem | ●○ | ●○ |  | ●○ |  |  | ●○ | ●○ |  |  |  | ● |
|  | Ertapenem |  | ●○ |  | ●○ |  |  |  | ●○ |  |  |  |  |
|  | Imipenem | ●○ | ●○ |  | ●○ |  |  | ●○ | ●○ |  |  |  | ●○ |
|  | Meropenem | ●○ | ●○ |  | ●○ |  |  | ●○ | ●○ |  |  |  | ●○ |
| Second-generation  cephalosporins | Cefoxitin |  |  |  |  |  |  |  |  |  |  | ●○ |  |
| Third-generation  cephalosporins | Ceftriaxone |  | ●○ | ●○ | ●○ | ●○ | ● |  | ●○ | ●○ | ●○ |  | ●○ |
|  | Ceftazidime |  | ●○ |  | ●○ |  |  | ●○ | ●○ | ●○ | ● |  |  |
|  | Cefotaxime |  | ●○ | ●○ | ●○ | ● | ● |  | ●○ | ●○ | ●○ |  | ●○ |
|  | Cefixime |  |  |  |  | ●○ |  |  |  |  |  |  |  |
| Fourth-generation  cephalosporins | Cefepime |  | ●○ |  | ●○ |  |  |  |  |  |  |  |  |
| Fluoroquinolones | Ciprofloxacin |  | ●○ |  | ●○ | ●○ | ● | ●○ | ●○ | ●○ | ●○ |  |  |
|  | Levofloxacin |  | ●○ | ●○ | ●○ |  | ● | ●○ | ●○ | ●○ | ● |  |  |
|  | Moxifloxacin |  | ●○ |  | ●○ |  |  |  | ● | ●○ | ● |  |  |
| Macrolides | Azithromycin |  |  |  |  | ●○ |  |  |  | ● | ●○ |  |  |
|  | Erythromycin |  |  |  |  |  |  |  |  |  |  | ●○ | ●○ |
| Penicillin | Amoxicillin-clavulanic acid |  |  | ●○ |  |  |  |  |  |  |  |  |  |
|  | Ampicillin |  | ●○ | ●○ |  |  |  |  |  | ●○ |  |  |  |
|  | Oxacillin |  |  |  |  |  |  |  |  |  |  | ●○ | ●○ |
|  | Penicillin G |  |  |  |  | ●○ | ● |  |  |  |  |  | ●○ |
|  | Mecillinam |  | ● |  | ● |  |  |  |  |  |  |  |  |
|  | Piperacillin/Tazobactam |  |  |  |  |  |  | ●○ |  |  |  |  |  |
| Polymyxin | Colistin | ●○ | ●○ |  | ●○ |  |  | ●○ |  |  |  |  |  |
| Sulfonamides | Co-trimoxazole |  | ●○ | ●○ | ●○ |  |  |  | ●○ | ●○ | ●○ |  | ●○ |
| Tetracyclines | Tigecycline | ●○ |  |  |  |  |  |  |  |  |  |  |  |
|  | Minocycline | ● |  |  |  |  |  |  |  |  |  |  |  |
| Others | Chloramphenicol |  |  |  |  |  |  |  |  | ●○ |  |  |  |
|  | Clindamycin |  |  |  |  |  |  |  |  |  |  | ●○ |  |
|  | Fosfomycin |  | ●○ |  | ●○ |  |  |  |  |  |  |  |  |
|  | Nitrofurantoin |  | ●○ |  |  |  |  |  |  |  |  |  |  |
|  | Rifampicin |  |  |  |  |  | ● |  |  |  |  |  |  |
|  | Spectinomycin |  |  |  |  | ●○ |  |  |  |  |  |  |  |
|  | Vancomycin |  |  |  |  |  |  |  |  |  |  | ●○ |  |

Pathogen-antimicrobial combinations: ● Included in GLASS-AMR 2.0 manual; ● Additional combinations included in the review; ○ Data available in the studies included in the review.

# Table S5. Summary of individual reports included in the review

| First author, Year of publication | Study design | Setting | Year of data collection | Location | Age group | Sex | Syndrome/Population | Specimen type | GLASS organisms included (N isolates) | Antibiotic sensitivity testing | |
| --- | --- | --- | --- | --- | --- | --- | --- | --- | --- | --- | --- |
|  |  |  |  |  |  |  |  |  |  | Phenotypic methods | Interpretative criteria (version) |
| Adhima, 2022^1^ | Retrospective | Hospital | 2019 | Surabaya | Adults and children | M+F | UTI | Urine | *A. baumannii* (5)  *E. coli* (40)  *K. pneumoniae* (12)  *P. aeruginosa* (14)  *S. aureus* (3)  *S. pneumoniae* (2) | Automated system (Phoenix, Vitek), microdilution  Reported MIC | CLSI (NS) |
| Afifurrahman, 2014^2^ | Cross-sectional | Hospital | 2012-2013 | Palembang | NS | NS | NS | Blood, exudative/transudative fluids, lower respiratory tract, pus, urine, swabs, others | *S. aureus* (1118) | NS | NS |
| Akbar, 2014^3^ | Retrospective | Hospital | 2018 | Pekanbaru | Adults | M+F | Diabetic ulcers | Wound swab | *A. baumannii (8)*  *K. pneumoniae (6)*  *E. coli (4)*  *P. aeruginosa (1)* | NS | NS |
| Amanda, 2020^4^ | Cross-sectional | Hospital | 2018-2019 | Jakarta | Adults | M+F | CAP | Lower respiratory tract | *S. pneumoniae* (14) | Microdilution  Reported MIC | CLSI (M100-S29) |
| Anggraini, 2022^5^ | Cross-sectional | Hospital | 2015-2019 | Riau | Children | NS | Patients in neonatal ICU with a bacterial infection | Blood, lower respiratory tract, pus, urine, and others | *A. baumannii* (1263) | Automated system (Vitek 2) | NS |
| Ariana, 2020^6^ | Retrospective | Hospital | 2018 | Surabaya | NS | NS | UTI | NS | *E. coli* (149) | NS | NS |
| Artono, 2022^7^ | Retrospective | Hospital | 2019-2020 | Surabaya | Adults and children | M+F | Chronic suppurative otitis media patients who had undergone mastoidectomy | Ear swab | *E. coli* (1)  *P. aeruginosa* (15) | Disc diffusion | CLSI (NS) |
| Azwar, 2012^8^ | Cross-sectional | Hospital | 2010 | Aceh | NS | NS | Chronic cough | Lower respiratory tract | *K. pneumoniae* (11)  *P. aeruginosa* (5)  *S. aureus* (9) | NS | NS |
| Bukitwetan, 2007^9^ | Cross-sectional | Community | 2003-2005 | Jakarta | Children | NS | Diarrhoea | Stool/rectal swab | *Salmonella spp.* (56) | Disc diffusion | CLSI (M02-A6, M100-S11) |
| Chudlori, 2012^10^ | Retrospective | Hospital | 2012 | Surakarta | Adults and children | M+F | NS | Pus | *S. aureus* (16) | Disc diffusion | CLSI (NS) |
| Chung, 2011^11^ | Prospective | Hospital | 2008-2009 | Jakarta, Surabaya, Semarang | Adults | M+F | Hospital-acquired or ventilator-acquired pneumonia | Blood, tracheal or bronchial aspirates, sputum | *K. pneumoniae (4)* | Microdilution | CLSI |
| Deurenberg, 2009^12^ | Cross-sectional | Community | 2006 | Yogyakarta | NS | M+F | Patients in ENT outpatient clinic | Nasal swabs | *S. aureus* (62) | Microdilution | CLSI (M100-S12) |
| Devian, 2022^13^ | Retrospective | Hospital | 2021 | Bali | Adults and children | M+F | Patients in ICU receiving ventilator treatment | Lower respiratory tract, blood | *A. baumannii* (37)  *K. pneumoniae* (31)  *P. aeruginosa* (41) | NS | CLSI (NS) |
| Donegan, 2006^14^ | Cross-sectional | Community | 2004 | Bali | Adults | F | Female sex workers attending STI clinic | Urethral, cervical, pharyngeal and rectal swabs | *N. gonorrhoeae* (147) | Agar dilution, disc diffusion  Reported MIC | CLSI (M07-A10, M100-S15) |
| Dwipoerwantoro, 2005^15^ | Cross-sectional | Hospital | 2001-2002 | Jakarta | Children | M+F | Dysentry | Stool/rectal swab | *Shigella spp.* (24) | Chromogenic agar | NS |
| Fabian, 2020^16^ | Retrospective | Hospital | 2016 | Surabaya | Adults and children | NS | NS | Blood | *A. baumannii* (164)  *P. aeruginosa* (63) | NS | NS |
| Farida, 2013^17^ | Cross-sectional | Community | 2010 | Semarang | Adults and children | M+F | Population-based survey, among healthy persons | Nasopharyngeal swab | *K. pneumoniae* (54) | Automated system (Vitek 2) | NS |
| Farida, 2014^18^ | Cross-sectional | Community | 2010 | Semarang | Adults and children | M+F | Population-based survey among healthy persons | Nasopharyngeal swab | *S. pneumoniae* (142) | Disc diffusion, E-test | EUCAST (v2.0) |
| Fathin, 2022^19^ | Retrospective | Hospital | 2017-2018 | Medan | Adults and children | M+F | Pneumonia | Lower respiratory tract | *A. baumannii* (10)  *E. coli* (2)  *K. pneumoniae* (13)  *P. aeruginosa* (6) | NS | NS |
| Firdiana, 2016^20^ | Cross-sectional | Community | 2016 | Semarang | Adults | F | Female sex workers with endocervical discharge | Urethral, cervical, pharyngeal and rectal swabs | *N. gonorrhoeae* (26) | Disc diffusion | NS |
| Hananta, 2016^21^ | Cross-sectional | Community | 2014 | Bali, Jakarta, Yogyakarta | Adults | M+F+  Transwomen | Persons attending STI services | Urethral, cervical, pharyngeal and rectal swabs | *N. gonorrhoeae* (79) | Disc diffusion, E-test  Reported MIC | EUCAST (v3.1) |
| Harimurti, 2016^22^ | Cross-sectional | Community | 2012 | Jakarta | Adults | M+F | People living with HIV | Nasopharyngeal swab | *S. pneumoniae* (20) | Disc diffusion | CLSI (M100-S16) |
| Harimurti, 2021^23^ | Prospective | Community | 2015 | Banda Aceh, Banjarmasin, Bekasi, Gorontalo, Jakarta, Makassar, Medan, Palangkaraya, Surabaya | NS | M+F | Pre-travel screening before Hajj | Nasopharyngeal swab | *S. pneumoniae* (70) | Disc diffusion | CLSI (M100-S26) |
| Harnanik, 2005^24^ | Cross-sectional | Hospital | 2005 | Yogyakarta | NS | NS | NS | Urine | *E. coli* (20) | Disc diffusion | NS |
| Herdiyanti, 2019^25^ | Retrospective | Hospital | 2017 | Surabaya | Adults | M+F | UTI | Urine | *E. coli* (127)  *K. pneumoniae* (36) | NS | NS |
| Herwana, 2010^26^ | Cross-sectional | Community | 2005-2007 | Jakarta | Children | NS | Diarrhoea | Stool/rectal swab | *Shigella spp.* (57) | Disc diffusion | CLSI (M100-S11) |
| Hidayah, 2020^27^ | Cross-sectional | Hospital | 2017 | Bojonegoro | Adults | M+F | Hospitalised patients (internal medicine and surgery) | Rectal swab | *E. coli* (80) | Disc diffusion | NS |
| Indraningrat, 2022^28^ | Retrospective | Hospital | 2018 | Bali | Adults and children | M+F | NS | Blood, lower respiratory tract, pus, urine, and others | *A. baumannii* (5)  *E. coli* (15)  *K. pneumoniae* (8)  *S. aureus* (8) | NS | NS |
| Indri, 2015^29^ | Cross-sectional | Hospital | 2012 | Palembang | Children | M+F | UTI | Urine | *E. coli* (118)  *K. pneumonia* (56)  *P. aeruginosa* (35)  *S. aureus* (65) | NS | NS |
| Indriatmi, 2019^30^ | Cross-sectional | Community | 2018 | Jakarta | Adults | M+F | Persons attending sexual health clinic | Urethral, cervical, pharyngeal and rectal swabs | *N. gonorrhoeae* (35) | Automated system (Vitek 2), disc diffusion | CLSI (M100-S27) |
| Karmila, 2020^31^ | Retrospective | Hospital | 2016-2018 | Palembang | Children | M+F | Neonates with clinical sepsis | Blood | *S. aureus* (5) | Automated system (Vitek 2) | CLSI (M100-S24) |
| Kartasasmita, 2002^32^ | Cross-sectional | Community | 1999-2000 | Bandung | Children | M+F | Pneumonia | Nasopharyngeal swab | *S. pneumoniae* (120) | Disc diffusion | NS |
| Khalilullah, 2011^33^ | Cross-sectional | Hospital | 2010 | Aceh | NS | NS | UTI | Urine | *A. baumannii* (15)  *E. coli* (37)  *K. pneumoniae* (33)  *P. aeruginosa* (12)  *S. aureus* (4) | Disc diffusion | CLSI (NS) |
| Kitagawa, 2018^34^ | Retrospective | Hospital | 2014 | Surabaya | Adults and children | NS | UTI | Urine | *E. coli* (214)  *K. pneumoniae* (76)  *P. aeruginosa* (27) | Disc diffusion | CLSI (M02-A7) |
| Lestari, 2008^35^ | Cross-sectional | Hospital and community | 2001-2002 | Semarang and Surabaya | NS | NS | Screening of patients admitted to or discharged from hospital, their relatives, and patients visiting primary health centres | Rectal swab | *E. coli* (3284) | Disc diffusion | CLSI (M02-A7) |
| Lestari, 2010^36^ | Cross-sectional | Hospital and community | 2001-2002 | Surabaya and Semarang | Adults and children | M+F | Screening of patients admitted to or discharged from hospital, their relatives, and patients visiting primary health centres | Nasal swab | *S. aureus* (361) | Disc diffusion | CLSI (M02-A7) |
| Lestari, 2013^37^ | Cross-sectional | Hospital | 2011 | Jakarta | NS | M+F | Patients in ICU | Blood and lower respiratory tract | *A. baumannii* (20)  *E. coli* (3)  *K. pneumoniae* (9)  *P. aeruginosa* (19) | NS | NS |
| Lugito, 2017^38^ | Retrospective | Hospital | 2011-2015 | Banten | Adults and children | M+F | NS | Blood | *Salmonella enterica serovar Typhi/Paratyphi A.*(168) | Agar dilution, automated system (Vitek 2)  Reported MIC | CLSI (M100-S23) |
| Mayasari, 2017^39^ | Retrospective | Hospital | 2017 | Banjarmasin | Adults | M+F | NS | Lower respiratory tract and pleural fluid | *K. pneumoniae* (25) | Agar dilution, automated system (Vitek 2) | NS |
| Meriyani, 2021^40^ | Retrospective | Hospital | 2017-2019 | Bali | Adults and children | M+F | Patients in ICU | Blood, lower respiratory tract, pus, and urine | *A. baumannii* (106)  *E. coli* (40)  *K. pneumoniae* (68)  *P. aeruginosa* (33) | Disc diffusion | CLSI (NS) |
| Meta, 2014^41^ | Cross-sectional | Hospital | 2013-2014 | Pekanbaru | Adults | M+F | Diabetic ulcers | Wound swab | *S. aureus (6)* | NS | NS |
| Moehario, 2009^42^ | Cross-sectional | Hospital | 2002-2008 | Jakarta | NS | M+F | Patients in ICU or internal medicine ward | Blood | *E. coli* (29)  *K. pneumoniae* (67)  *Salmonella enterica serovar Typhi/Paratyphi A* (35)  *P. aeruginosa* (90) | Disc diffusion | CLSI (NS) |
| Moehario, 2020^43^ | Retrospective | Hospital | 2015-2018 | Jakarta | Adults | M+F | Hospitalised and ICU patients | Blood, lower respiratory tract, urine, throat swab, pus | *A. baumannii* (44) | Disc diffusion | CLSI (M100-S28) |
| Muktiarti, 2021^44^ | Prospective | Community | 2013 | Jakarta | Children | NS | People living with HIV on antiretroviral therapy | Nasopharyngeal swab | *S. pneumoniae* (27) | Disc diffusion | CLSI (M100-S29) |
| Murni, 2016^45^ | Prospective | Hospital | 2010-2013 | Yogyakarta | Children | M+F | Hospitalised with suspicion of nosocomial bloodstream infection | Blood | *E. coli* (1)  *K. pneumoniae* (9)  *P. aeruginosa* (93) | Disc diffusion | CLSI (M100-S21) |
| Nathania, 2022^46^ | Retrospective | Hospital | 2015-2021 | Jakarta | NS | NS | Typhoid fever | NS | *Salmonella enterica serovar Typhi/Paratyphi A* (28) | Disc diffusion | CLSI (M100-S21, M100-S30), EUCAST (v12.0) |
| Nirwati, 2018^47^ | Cross-sectional | Hospital | 2016-2017 | Klaten | Adults and children | M+F | Hospitalised patients | Blood, lower respiratory tract, pus, wound swab, and others | *A. baumannii* (80) | Disc diffusion | CLSI (M100-S25) |
| Nirwati, 2019^48^ | Retrospective | Hospital | 2017-2018 | Klaten | Adults and children | M+F | Hospitalised patients | Blood, lower respiratory tract, pus, stool, urine, and others | *K. pneumoniae* (167) | Disc diffusion | CLSI (M100-S25) |
| Nur, 2016^49^ | Cross-sectional | Hospital | 2015 | Aceh | Adults | M+F | Diabetic foot ulcers | Foot ulcer swab and pus | *E. coli* (24)  *Shigella spp.* (39) | Disc diffusion | CLSI (M100-S25) |
| Oyofo, 2002^50^ | Cross-sectional | Hospital | 1999-2000 | Bali, Batam, Jakarta, Makassar, Medan, Padang, Pontianak | Adults and children | M+F | Diarrhoea | Stool/rectal swab | *Salmonella spp.* (158)  *Shigella spp.* (277) | Disc diffusion | NS |
| Parut, 2015^51^ | Cross-sectional | Hospital | 2014 | Surabaya | Adults | F | Pregnant women with asymptomatic bacteriuria | Urine | *E. coli* (5)  *K. pneumoniae* (1) | Disc diffusion | CLSI (M31-A2) |
| Patilaya, 2019^52^ | Cross-sectional | Hospital | 2016 | Klaten | NS | NS | Post-surgical patients | Pus | *K. pneumoniae* (20) | Disc diffusion | CLSI (M100S) |
| Pelegrin, 2019^53^ | Prospective | Hospital | 2013 | Jakarta | Adults | NS | ICU patients | Blood, lower respiratory tract, rectal swab, throat swab, tissue swab, urine, wound swab | *P. aeruginosa* (107) | Automated system (Vitek 2)  Reported MIC | EUCAST (v8.1) |
| Permana, 2022^54^ | Cross-sectional | Hospital | 2020 | Bandung | Adults | M+F | Diabetic foot infection | Wound | *A. baumannii* (8)  *E. coli* (7)  *K. pneumoniae* (13)  *S. aureus* (6) | Automated system (Vitek 2) | NS |
| Prabowo, 2012^55^ | Cross-sectional | Hospital | 2011 | Yogyakarta | Adults | M+F | UTI | Urine | *E. coli* (18)  *Salmonella enterica serovar Typhi/Paratyphi A* (1) | Disc diffusion | CLSI (NS) |
| Punjabi, 2013^56^ | Prospective | Community | 2001-2003 | Jakarta | All | M+F | Diarrhoea or fever | Blood | *Salmonella enterica serovar Typhi/Paratyphi A* (262) | Disc diffusion | NS |
| Purba, 2019^57^ | Retrospective | Hospital | 2014-2016 | Surabaya | Adults | M+F | CAP | Blood and lower respiratory tract | *A. baumannii* (27)  *E. coli* (10)  *K. pneumoniae* (25)  *P. aeruginosa* (19)  *S.aureus* (9)  *S. pneumoniae* (26) | Disc diffusion | CLSI (NS) |
| Puspandari, 2016^58^ | Retrospective | Community | 2016 | Jakarta | Adults | F | Female sex workers | Urethral, cervical, pharyngeal and rectal swabs | *N. gonorrhoeae* (179) | Automated system (Vitek 2), disc diffusion | CLSI (M100-S25) |
| Rachman, 2016^59^ | Cross-sectional | Hospital | 2015 | Banjarmasin | NS | NS | Diabetic mellitus with UTI | Urine | *E. coli* (17)  *P. aeruginosa* (3) | Disc diffusion | NS |
| Radji, 2011^60^ | Cross-sectional | Hospital | 2009-2010 | Jakarta | NS | NS | Patients in ICU treated receiving antibiotics | Blood, peritoneal fluid, respiratory tract, urine, and wound | *E. coli* (13)  *K. pneumoniae* (38)  *P. aeruginosa* (66) | Disc diffusion | CLSI (M100-S16) |
| Radji, 2014^61^ | Retrospective | Hospital | 2012 | Jakarta | Adults | M+F | Patients hospitalized with diabetic foot | Foot ulcer samples | *E. coli (6)*  *S. aureus* (35) | Disc diffusion | CLSI (M100-S16) |
| Rahman, 2015^62^ | Retrospective | Hospital | 2014 | Riau | NS | NS | Patients in ICU | Blood, lower respiratory tract | *A. baumannii* (59) | Automated system (Vitek 2) | NS |
| Ramdhani, 2022^63^ | Retrospective | Hospital | 2017 | Tasikmalaya | Adults | NS | Acute respiratory tract infection | Throat swab | *H. influenzae* (77) | Disc diffusion | NS |
| Rosana, 2020a^64^ | Cross-sectional | Community | 2015-2017 | Jakarta | Adults and children | F | Pregnant women with no symptoms of UTI | Urine | *E. coli* (20)  *K. pneumoniae* (15) | Automated system (Vitek) | CLSI (M100-S25) |
| Rosana, 2020b^65^ | Cross-sectional | Hospital and community | 2014-2015 | Jakarta | NS | NS | UTI | Urine | *E. coli* (16)  *K. pneumoniae* (11) | Automated system (Vitek 2) | CLSI (NS) |
| Rosyid, 2021^66^ | Cross-sectional | Community | 2019 | Surabaya | Adults | M+F | Lower respiratory tract infection | Lower respiratory tract | *A. baumannii* (4)  *K. pneumoniae* (16)  *P. aeruginosa* (8)  *S. aureus* (11)  *S. pneumoniae* (3) | Automated system (Vitek 2), microdilution  Reported MIC | CLSI (NS) |
| Safari, 2014^67^ | Cross-sectional | Community | 2012 | Jakarta | Children | M+F | Screening of people living with HIV | Nasopharyngeal swab | *S. pneumoniae* (42) | Disc diffusion | CLSI (M100-S17) |
| Safari, 2015^68^ | Cross-sectional | Community | 2011 | Jakarta | Adults | M+F | Screening at routine clinics | Nasopharyngeal swab | *S. aureus* (42)  *S. pneumoniae* (4) | Disc diffusion | CLSI (M100-S17) |
| Safari, 2020^69^ | Retrospective | Hospital | 2012 | Jakarta | Adults and children | NS | People living with HIV | Nasopharyngeal swab | *H. influenzae* (34) | Disc diffusion | CLSI (NS) |
| Safari, 2022a^70^ | Retrospective | Hospital | 2017 | Bali | Adults and children | NS | Fever and upper respiratory tract symptoms | Nasopharyngeal swab | *S. pneumoniae* (37) | Disc diffusion | CLSI (M100-S29) |
| Safari, 2022b^71^ | Cross-sectional | Community | 2018-2019 | Banyumas | Children | NS | Acute otitis media | Nasopharyngeal swab | *H. influenzae* (85) | Microdilution  Reported MIC | CLSI (M100-S29) |
| Sahara, 2023^72^ | Retrospective | Hospital | 2018 | Lampung | NS | NS | Hospitalised patients | Blood, urine, pus, sputum, bodily fluids, swabs | *E. coli* (1) | Disc diffusion  Automated system (Vitek 2) | CLSI (M100-S20) |
| Saharman, 2018^73^ | Prospective | Hospital | 2013-2014 | Jakarta | Adults | M+F | Patients in ICU | Stool/rectal swabs, throat swabs | *A. baumannii* (325) | Automated system (Vitek 2), MALDI-TOF MS  Reported MIC | EUCAST (v3.1) |
| Saharman, 2019^74^ | Prospective | Hospital | 2013-2014 | Jakarta | Adults | M+F | Patients in ICU | Stool/rectal swabs, throat swabs | *P. aeruginosa* (281) | Automated system (Vitek 2), MALDI-TOF MS  Reported MIC | EUCAST (v3.1) |
| Saharman, 2020^75^ | Prospective | Hospital | 2013-2014 | Jakarta | Adults | M+F | Patients in ICU | Stool/rectal swabs, throat swabs | *K. pneumoniae* (370) | Automated system (Vitek 2), MALDI-TOF MS  Reported MIC | EUCAST (v3.1) |
| Said, 2017^76^ | Cross-sectional | Hospital | 2014 | Jakarta | Adults | M+F | Various, including tuberculosis and pneumonia | Lower respiratory tract | *S. pneumoniae* (13) | Disc diffusion  Reported MIC | CLSI (M100-S25) |
| Salsabila, 2021^77^ | Cross-sectional | Community | 2019 | Kotabaru | Children | M+F | Screening at community health services | Nasopharyngeal swab | *S. pneumoniae* (85) | Disc diffusion, microdilution  Reported MIC | CLSI (M100-S29) |
| Santoso, 2022^78^ | Retrospective | Hospital | 2021-2022 | Bandung | Adults | M+F | COVID-19 with secondary pulmonary bacterial infection | Lower respiratory tract | *A. baumannii* (58)  *K. pneumoniae* (36)  *P. aeruginosa* (16)  *S. aureus* (5) | Automated system (Vitek 2) | CLSI (M60-Ed2, M100-S30) |
| Saputro, 2013^79^ | Cross-sectional | Community | 2013 | Semarang | Children | M+F | Pneumonia | Nasopharyngeal swab | *S. pneumoniae* (23) | Disc diffusion | CLSI (M100-S22) |
| Sari, 2015^80^ | Retrospective | Hospital | 2013 | Palembang | Children | M+F | Patients in neonatal ICU with a bacterial infection | Blood, lower respiratory tract, urine | *E. coli* (9)  *K. pneumoniae* (12)  *P. aeruginosa* (9)  *S. aureus* (7) | NS | NS |
| Sari, 2017^81^ | Cross-sectional | Hospital | 2014-2015 | Central Java | Children | M+F | Patients in neonatal ICU with a bacterial infection | Blood | *A. baumannii* (21)  *K. pneumoniae* (49)  *P. aeruginosa* (31) | Agar dilution, automated system (Vitek 2), disc diffusion  Reported MIC | NS |
| Setyati, 2012^82^ | Retrospective | Hospital | 2011 | Yogyakarta | Children | NS | Patients in paediatric ICU with pneumonia | Blood and tracheal aspirate | *E. coli* (3)  *K. pneumoniae* (15)  *P. aeruginosa* (37)  *S. aureus* (2) | Disc diffusion | NS |
| Sholeh, 2020^83^ | Cross-sectional | Hospital | 2017 | Surabaya | Adults and children | M+F | Patients in ICU or infectious diseases ward | Rectal swab | *E. coli* (64) | Disc diffusion | CLSI (M100-S26) |
| Sinto, 2022^84^ | Retrospective | Hospital and community | 2019-2020 | Jakarta | Adults and children | M+F | Hospitalised patients | Blood | *E. coli* (230)  *K. pneumoniae* (408)  *P. aeruginosa* (157)  *Salmonella spp.* (33)  *S. aureus* (216)  *S. pneumoniae* (3) | Automated system (Vitek 2), disc diffusion | CLSI (M39-A4) |
| Soedarmono, 2022^85^ | Prospective | Hospital | 2013-2016 | Bandung, Denpasar, Jakarta,  Makassar, Semarang, Surabaya, Yogyakarta | Adults and children | M+F | Fever | Blood | *E. coli* (14)  *K. pneumoniae* (5)  *P. aeruginosa* (2)  *Salmonella enterica serovar Typhi/Paratyphi A* (51)  *S. aureus* (10)  *S. pneumoniae* (2) | Automated system (Phoenix, Vitek 2) | NS |
| Subagdja, 2022^86^ | Retrospective | Hospital | 2020-2021 | Bandung | Adults | M+F | COVID-19 | Blood, lower respiratory tract, pus, and urine | *A. baumannii* (134)  *E. coli* (69)  *K. pneumoniae* (148)  *P. aeruginosa* (65)  *S. aureus* (15) | Automated system (Vitek 2) | CLSI (M100-S30) |
| Sugianli, 2017^87^ | Cross-sectional | Hospital and community | 2014-2015 | Bandung and Medan | Adults | M | UTI | Urine | *E. coli* (521)  *K. pneumonia* (136) | Disc diffusion | CLSI (M02-A11), EUCAST (NS) |
| Suranadi, 2021^88^ | Prospective | Hospital | 2018-2021 | Bali | Adults | M+F | Patients in ICU and high care unit | Blood, lower respiratory tract, pleural fluid, pus, tissue swab, urine, wound swab | *P. aeruginosa* (135) | Automated system (Vitek 2) | CLSI (M100-S22) |
| Sutrisna, 2006^91^ | Cross-sectional | Community | 2004 | Papua, Surabaya | Adults | M+F | Sex workers | Urethral, cervical, pharyngeal and rectal swabs | *N. gonorrhoeae* (163) | Dilution methods | NS |
| Tan, 2021^92^ | Prospective | Hospital | 2019-2020 | Surabaya | Adults | M+F | Hospitalised people living with HIV | Blood and lower respiratory tract | *A. baumannii* (4)  *E. coli* (5)  *K. pneumoniae* (14)  *P. aeruginosa* (4)  *S. aureus* (4)  *Salmonella spp.* (2) | NS | NS |
| Tandiono, 2022^93^ | Retrospective | Hospital | 2019-2020 | Tangerang | Adults and children | M+F | Patients in ICU | Blood, CSF, lower respiratory tract, pus, stool, urine, and others | *E. coli* (89) | Automated system (Vitek 2) | NS |
| Tauran, 2022^94^ | Retrospective | Hospital and community | 2015-2018 | Makassar | Adults and children | M+F | Hospitalised patients | Blood | *E. coli* (104)  *K. pneumoniae* (171)  *P. aeruginosa* (68)  *S. aureus* (245) | Automated system (Vitek 2), disc diffusion | CLSI (NS) |
| Tjaniadi, 2003^96^ | Cross-sectional | Hospital | 1995-2001 | Batam, Denpasar, Jakarta, Makassar Medan, Padang, Pontianak, | Adults and children | M+F | Diarrhoea | Stool/rectal swab | *Shigella spp.* (767) | Disc diffusion | CLSI (M02-A6) |
| Tjoa, 2013^97^ | Cross-sectional | Hospital | 2010-2011 | Jakarta | Children | NS | Sepsis and other neonates in ICU | Blood, skin swabs | *A. baumannii (17)* | Disc diffusion | CLSI |
| Wang, 2011^98^ | Cross-sectional | Hospital | 2009-2010 | Semarang | Adults | NS | CAP | Blood, lower respiratory tract, middle ear fluid, nasopharyngeal swabs, and pleural fluid | *K. pneumoniae* (41)  *S. pneumoniae* (55) | Microdilution  Reported MIC | CLSI (M45-H, M100-S20) |
| Wardhana, 2017^99^ | Retrospective | Hospital | 2016 | Jakarta | Adults and children | M+F | Burns | Lower respiratory tract, blood, tissue, wound swab and urine | *A. baumannii* (10)  *K. pneumoniae* (15)  *P. aeruginosa* (11) | NS | NS |
| Wijaksana, 2019^100^ | Retrospective | Hospital | 2017 | Riau | Adults | M+F | Patients in ICU with sepsis | Lower respiratory tract, blood, urine, CSF | *A. baumannii* (14)  *E. coli* (5)  *K. pneumoniae* (9)  *P. aeruginosa* (6) | E-test | NS |
| Wikaningtyas, 2015^101^ | Cross-sectional | Hospital | 2012 | Bandung | NS | M+F | Patients in ICU | Lower respiratory tract and others | *A. baumannii* (12)  *E. coli* (36)  *K. pneumoniae* (10)  *S. aureus* (11) | Disc diffusion | NS |
| Yanagi, 2009^102^ | Retrospective | Hospital | 2006 | Surabaya | NS | NS | Enteric fever | Blood and stool | *Salmonella enterica serovar Typhi/Paratyphi A* (17) | Disc diffusion | CLSI (M100-S18) |

Abbreviations: CAP, Community-acquired pneumonia; CLSI, Clinical and Laboratory Standards Institute; ENT, ear, nose, and throat; EUCAST, European Committee on Antimicrobial Susceptibility Testing; F, female; ICU, intensive care unit; M, male; MALDI-TOF MS, matrix-assisted laser desorption/ionization coupled with time-of-flight mass spectrometry; NS, not specified; STI, sexually transmitted infection; UTI, urinary tract infection

# Table S6. Summary of number of bacterial isolates and specimen types included in the review for each GLASS pathogen

| Microorganism | Number of studies | Number of isolates | | | | | | | | | | | | | | | |
| --- | --- | --- | --- | --- | --- | --- | --- | --- | --- | --- | --- | --- | --- | --- | --- | --- | --- |
|  |  | **Hospital** | | | | | | | | **Community** | | | | | | | |
|  |  | **Blood** | **CSF** | **LRT** | **Stool** | **Urethral, cervical,**  **pharyngeal and rectal**  **swabs** | **Urine** | **Other** | **Total** | **Blood** | **CSF** | **LRT** | **Stool** | **Urethral, cervical,**  **pharyngeal and rectal**  **swabs** | **Urine** | **Other** | **Total** |
| *A. baumannii* | 26 | 578 | 0 | 69 |  |  |  | 2128 | 2775 | 53 | 0 | 4 |  |  |  | 0 | 57 |
| *E. coli* | 40 | 384 | 0 | 15 |  |  | 837 | 1417 | 2653 | 123 | 0 | 0 |  |  | 307 | 2500 | 2930 |
| *H. influenzae* | 3 | 0 | 0 | 0 |  |  |  | 107 | 107 | 0 | 0 | 0 |  |  |  | 85 | 85 |
| *K. pneumoniae* | 41 | 931 | 0 | 101 |  |  | 331 | 1010 | 2373 | 97 | 0 | 16 |  |  | 80 | 54 | 247 |
| *P. aeruginosa* | 31 | 498 | 0 | 109 |  |  |  | 879 | 1486 | 60 | 0 | 8 |  |  |  | 0 | 68 |
| *N. meningitidis* | 0 | 0 | 0 |  |  |  |  | 0 | 0 | 0 | 0 |  |  |  |  | 0 | 0 |
| *N. gonorrhoeae* | 6 |  |  |  |  | 0 |  | 0 | 0 |  |  |  |  | 629 |  | 0 | 629 |
| *Salmonella spp.* (non-typhoidal) | 3 | 18 |  |  | 158 |  |  | 0 | 176 | 27 |  |  | 56 |  |  | 0 | 83 |
| *Salmonella enterica* serovar Typhi and Paratyphi A | 8 | 254 |  |  | 0 |  |  | 46 | 300 | 262 |  |  | 0 |  |  | 0 | 262 |
| *Shigella spp.* | 5 |  |  |  | 1068 |  |  | 39 | 1107 |  |  |  | 57 |  |  | 0 | 57 |
| *S. aureus* | 25 | 786 | 5 | 430 |  |  |  | 1286 | 2507 | 132 | 0 | 11 |  |  |  | 673 | 816 |
| *S. pneumoniae* | 18 | 5 | 0 | 27 |  |  |  | 131 | 163 | 0 | 0 | 3 |  |  |  | 633 | 636 |

Specimen types were classified according to the GLASS priority specimen type categories.

Abbreviations: CSF, cerebrospinal fluid; LRT, lower respiratory tract

**Figure S1.** AMR prevalence estimates for all GLASS-specific pathogen-antimicrobial combinations in hospital and community settings.

Figure shows bar charts of AMR prevalence estimates for GLASS-specific pathogen-antimicrobial combinations, for hospitals (dark color) and communities (light color), accounting for sampling weights of the individual studies. 95% confidence intervals were estimated using Wilson score interval (where the number of studies was greater than one).

^a^The estimate for third-generation cephalosporins is derived only from ceftazidime.

^b^Resistance to methicillin detected using either cefoxitin or oxacillin.

^c^The estimate for fluoroquinolones is derived only from ciprofloxacin.

^d^The estimate for fluoroquinolones is derived only from levofloxacin.

# Table S7. Numbers and proportions of resistant isolates for *Klebsiella pneumoniae*-antimicrobial combinations

| Antibiotics | Hospitals | | | | | Communities | | | | |
| --- | --- | --- | --- | --- | --- | --- | --- | --- | --- | --- |
|  | **Blood** | **Urine** | **Lower respiratory tract** | **Other** | **Total** | **Blood** | **Urine** | **Lower respiratory tract** | **Other** | **Total** |
| Aminoglycosides |  |  |  |  |  |  |  |  |  |  |
| Amikacin | 199/890 (23.0%; 20.4-25.9) [7] | 34/265 (14.3%; 10.6-19.0) [5] | 13/174 (14.4%; 10.0-20.4) [4] | 90/623 (15.0%; 12.4-18.0) [16] | 336/1952 (18.8%; 17.1-20.6) [31] | 5/32 (15.6%) [1] | 0/19 (0.0%) [1] |  | 12/61 (19.7%) [1] | 17/112 (11.9%; 7.1-19.2) [3] |
| Gentamicin | 642/882 (72.8%; 69.8-75.6) [7] | 102/206 (50.8%; 44.1-57.6) [4] | 39/90 (43.3%; 33.6-53.6) [4] | 220/593 (36.9%; 33.1-40.9) [14] | 1003/1771 (57.3%; 55.0-59.6) [28] | 21/32 (65.6%) [1] | 3/34 (12.5%; 5.1-27.5) [2] | 0/16 (0.0%) [1] | 26/56 (46.4%) [1] | 50/138 (37.2%; 29.6-45.5) [5] |
| Carbapenems | 23.0% (20.1-26.2) [3] | 34.7% (28.8-41.0) [4] | 19.3% (12.4-28.8) [4] | 23.9% (20.3-27.8) [9] | 21.6% (19.5-23.9) [18] | 29.6% [1] | 0.0% [1] | 6.3% (3.3-11.5) [3] | 26.9% [1] | 28.3% (19.2-39.6) [2] |
| Doripenem | 113/623 (21.4%; 18.4-24.8) [3] |  |  | 5/25 (20.0%; 8.9-39.1) [2] | 118/648 (21.4%; 18.4-24.7) [5] | 8/27 (29.6%) [1] |  |  | 10/40 (25.0%) [1] | 18/67 (27.5%; 18.3-39.2) [2] |
| Ertapenem | 8/80 (19.2%; 12.1-29.2) [2] | 25/85 (29.4%) [1] | 17/77 (22.1%; 14.3-32.5) [3] | 13/124 (8.8%; 5.0-15.1) [4] | 63/366 (20.0%; 16.3-24.4) [10] |  |  |  |  |  |
| Imipenem | 143/714 (23.0%; 20.0-26.2) [6] | 9/144 (6.3%; 3.3-11.5) [3] | 2/13 (15.4%) [1] | 118/493 (23.9%; 20.3-27.8) [9] | 272/1364 (21.6%; 19.5-23.9) [18] | 8/27 (29.6%) [1] |  |  | 12/45 (26.7%) [1] | 20/72 (28.3%; 19.2-39.6) [2] |
| Meropenem | 157/729 (23.0%; 20.1-26.2) [3] | 86/229 (34.7%; 28.8-41.0) [4] | 17/88 (19.3%; 12.4-28.8) [4] | 149/983 (15.4%; 13.3-17.8) [16] | 409/2029 (20.4%; 18.7-22.2) [27] | 8/32 (25.0%) [1] | 0/15 (0.0%) [1] | 1/16 (6.3%) [1] | 14/52 (26.9%) [1] | 23/115 (21.2%; 14.7-29.5) [4] |
| Third generation  cephalosporins | 86.9% (84.5-89.0) [7] | 89.1% (84.6-92.4) [3] | 67.8% (57.6-76.5) [4] | 52.3% (47.4-57.2) [12] | 74.4% (72.3-76.4) [27] | 70.3% (59.7-79.1) [2] | 67.9% [1] | 6.3% [1] | 1.9% [1] | 53.5% (46.0-60.9) [5] |
| Ceftriaxone | 752/866 (86.9%; 84.5-89.0) [7] | 224/248 (89.1%; 84.6-92.4) [3] | 61/90 (67.8%; 57.6-76.5) [4] | 308/596 (51.6%; 47.6-55.6) [14] | 1345/1800 (74.4%; 72.3-76.4) [27] | 54/82 (65.1%; 54.3-74.5) [2] | 38/56 (67.9%) [1] | 0/16 (0.0%) [1] | 1/54 (1.9%) [1] | 93/208 (45.8%; 39.2-52.6) [5] |
| Ceftazidime | 422/763 (59.1%; 55.6-62.5) [6] | 263/304 (84.5%; 80.0-88.1) [4] | 56/99 (55.7%; 45.9-65.1) [5] | 205/428 (47.8%; 43.2-52.6) [13] | 946/1594 (59.7%; 57.2-62.0) [27] | 52/81 (62.4%; 51.5-72.1) [2] | 37/71 (52.1%; 40.7-63.3) [2] | 1/16 (6.3%) [1] |  | 90/168 (53.5%; 46.0-60.9) [5] |
| Cefotaxime | 642/798 (81.5%; 78.7-84.0) [7] | 123/177 (69.5%; 62.4-75.8) [4] | 46/75 (60.9%; 49.6-71.2) [4] | 206/393 (52.3%; 47.4-57.2) [12] | 1017/1443 (71.8%; 69.5-74.1) [26] | 58/82 (70.3%; 59.7-79.1) [2] | 1/15 (6.7%) [1] |  | 1/54 (1.9%) [1] | 60/151 (42.3%; 34.7-50.3) [4] |
| Fourth generation  cephalosporins |  |  |  |  |  |  |  |  |  |  |
| Cefepime | 557/668 (83.3%; 80.3-85.9) [6] | 174/192 (89.8%; 84.7-93.3) [2] | 44/90 (48.9%; 38.8-59.0) [4] | 220/523 (42.2%; 38.0-46.4) [11] | 995/1473 (68.9%; 66.5-71.2) [22] | 24/41 (58.5%) [1] | 33/71 (46.5%; 35.4-58.0) [2] |  |  | 57/112 (52.2%; 43.1-61.3) [3] |
| Fluoroquinolones | 53.9% (50.3-57.5) [7] | 77.5% (69.6-83.8) [3] | 55.8% (42.3-68.4) [2] | 52.3% (40.4-64.0) [2] | 53.1% (50.5-55.7) [28] | 67.3% [1] | 50.7% (39.3-62.0) [2] | 12.5% [1] |  | 53.8% (45.5-61.9) [4] |
| Ciprofloxacin | 410/753 (53.9%; 50.3-57.5) [7] | 100/130 (77.5%; 69.6-83.8) [3] | 44/90 (48.9%; 38.8-59.0) [4] | 229/488 (45.7%; 41.3-50.1) [15] | 783/1461 (53.1%; 50.5-55.7) [28] | 35/52 (67.3%) [1] | 36/71 (50.7%; 39.3-62.0) [2] | 2/16 (12.5%) [1] |  | 73/139 (53.8%; 45.5-61.9) [4] |
| Levofloxacin | 237/810 (30.8%; 27.7-34.1) [5] | 94/173 (55.7%; 48.2-62.9) [3] | 29/52 (55.8%; 42.3-68.4) [2] | 115/362 (31.7%; 27.1-36.6) [10] | 475/1397 (35.2%; 32.7-37.7) [20] | 19/77 (25.8%; 17.3-36.6) [2] | 32/71 (45.1%; 34.0-56.6) [2] |  |  | 51/148 (33.9%; 26.8-41.9) [4] |
| Moxifloxacin |  |  |  | 34/65 (52.3%; 40.4-64.0) [2] | 34/65 (52.3%; 40.4-64.0) [2] |  |  |  |  |  |
| Polymyxin |  |  |  |  |  |  |  |  |  |  |
| Colistin |  |  |  | 46/52 (88.5%; 77.0-94.6) [3] | 46/52 (88.5%; 77.0-94.6) [3] |  |  |  |  |  |
| Sulfonamides |  |  |  |  |  |  |  |  |  |  |
| Co-trimoxazole | 355/537 (60.4%; 56.2-64.4) [4] | 126/156 (81.4%; 74.6-86.7) [5] | 36/77 (46.8%; 36.0-57.8) [3] | 250/529 (45.3%; 41.1-49.6) [12] | 767/1299 (57.3%; 54.6-60.0) [24] |  | 12/43 (41.8%; 28.3-56.6) [3] | 1/16 (6.3%) [1] | 15/24 (62.5%) [1] | 28/83 (45.4%; 35.2-56.1) [5] |
| Other antibiotics |  |  |  |  |  |  |  |  |  |  |
| Fosfomycin | 257/331 (77.6%; 72.9-81.8) [3] | 43/168 (21.9%; 16.3-28.7) [4] | 1/13 (7.7%) [1] | 6/63 (8.1%; 3.5-17.5) [4] | 307/575 (47.4%; 43.3-51.5) [11] |  | 3/30 (8.1%; 2.5-23.2) [2] |  |  | 3/30 (8.1%; 2.5-23.2) [2] |

The data are presented as: number of resistant isolates (numerator)/number of total isolates (denominator) (AMR prevalence estimates; 95%CI) [N studies]. 95% confidence intervals were estimated using Wilson score interval where the number of studies was greater than one.

# Table S8. Numbers and proportions of resistant isolates for *Escherichia coli*-antimicrobial combinations

| Antibiotics | Hospitals | | | | | Communities | | | |
| --- | --- | --- | --- | --- | --- | --- | --- | --- | --- |
|  | **Blood** | **Urine** | **Lower respiratory tract** | **Other** | **Total** | **Blood** | **Urine** | **Other** | **Total** |
| Aminoglycosides |  |  |  |  |  |  |  |  |  |
| Amikacin | 33/357 (9.6%; 7.0-13.1) [5] | 62/740 (8.3%; 6.5-10.5) [5] | 1/5 (28.9%; 6.8-69.3) [2] | 164/847 (22.3%; 19.6-25.2) [20] | 260/1949 (13.8%; 12.3-15.4) [32] | 15/117 (12.9%; 8.0-20.2) [2] | 26/280 (9.3%) [1] |  | 41/397 (10.4%; 7.8-13.8) [3] |
| Gentamicin | 136/352 (38.9%; 33.9-44.1) [5] | 310/685 (45.1%; 41.4-48.9) [7] | 1/5 (28.9%; 6.8-69.3) [2] | 418/1497 (27.0%; 24.8-29.3) [17] | 865/2539 (34.3%; 32.5-36.2) [31] | 31/113 (27.5%; 20.1-36.3) [2] | 89/300 (29.7%; 24.8-35.1) [2] | 61/2500 (2.4%) [1] | 181/2913 (6.3%; 5.5-7.2) [5] |
| Carbapenems | 9.8% (6.8-13.9) [4] | 8.2% (5.9-11.4) [4] | 100.0% [1] | 40.2% (34.8-45.8) [9] | 18.3% (16.0-20.9) [18] | 15.7% [1] | 3.3% (1.8-6.0) [2] |  | 15.7% [1] |
| Doripenem | 14/256 (6.4%; 4.0-10.1) [3] |  |  | 4/36 (11.1%) [1] | 18/292 (6.9%; 4.5-10.4) [4] | 10/80 (12.5%) [1] |  |  | 10/80 (12.5%) [1] |
| Ertapenem | 1/22 (4.5%) [1] | 68/242 (24.1%; 19.1-29.8) [2] |  | 121/372 (61.3%; 56.3-66.2) [7] | 190/636 (31.8%; 28.3-35.5) [10] |  | 26/280 (9.3%) [1] |  | 26/280 (9.3%) [1] |
| Imipenem | 25/278 (9.8%; 6.8-13.9) [4] | 32/390 (8.2%; 5.9-11.4) [4] | 1/3 (33.3%) [1] | 123/305 (40.2%; 34.8-45.8) [9] | 181/976 (18.3%; 16.0-20.9) [18] | 13/83 (15.7%) [1] |  |  | 13/83 (15.7%) [1] |
| Meropenem | 25/329 (8.1%; 5.6-11.5) [4] | 14/463 (3.0%; 1.8-5.0) [5] | 2/2 (100.0%) [1] | 141/761 (24.8%; 21.9-28.0) [18] | 182/1555 (13.9%; 12.3-15.7) [28] | 12/105 (11.6%; 6.8-19.2) [2] | 10/300 (3.3%; 1.8-6.0) [2] |  | 22/405 (5.8%; 3.9-8.5) [4] |
| Third generation  cephalosporins | 78.7% (73.8-82.8) [5] | 77.0% (73.6-80.1) [5] | 100.0% [1] | 55.8% (52.4-59.1) [19] | 66.4% (64.0-68.6) [31] | 68.0% [1] | 55.0% [1] | 1.2% [1] | 58.1% (53.1-62.9) [3] |
| Ceftriaxone | 265/354 (75.1%; 70.4-79.3) [6] | 322/435 (73.7%; 69.3-77.6) [5] | 2/2 (100.0%) [1] | 470/834 (55.8%; 52.4-59.1) [19] | 1059/1625 (66.4%; 64.0-68.6) [31] | 68/105 (65.0%; 55.5-73.5) [2] | 154/280 (55.0%) [1] |  | 222/385 (58.1%; 53.1-62.9) [3] |
| Ceftazidime | 246/312 (78.7%; 73.8-82.8) [5] | 495/642 (77.0%; 73.6-80.1) [5] | 2/3 (66.7%) [1] | 397/802 (47.3%; 43.9-50.8) [17] | 1140/1759 (66.3%; 64.1-68.5) [28] | 56/95 (59.2%; 49.2-68.6) [2] | 149/300 (49.7%; 44.0-55.3) [2] |  | 205/395 (52.5%; 47.5-57.3) [4] |
| Cefotaxime | 223/317 (72.3%; 67.1-76.9) [5] | 218/311 (70.1%; 64.8-74.9) [4] | 4/5 (71.1%; 30.7-93.2) [2] | 405/1374 (29.5%; 27.2-32.0) [19] | 850/2007 (43.6%; 41.4-45.7) [30] | 66/97 (68.0%) [1] | 1/20 (5.0%) [1] | 31/2500 (1.2%) [1] | 98/2617 (4.1%; 3.4-4.9) [3] |
| Fourth generation  cephalosporins |  |  |  |  |  |  |  |  |  |
| Cefepime | 170/244 (73.3%; 67.4-78.4) [4] | 228/301 (75.5%; 70.4-80.0) [3] | 2/5 (13.3%; 1.8-56.8) [2] | 360/722 (50.8%; 47.1-54.4) [12] | 760/1272 (63.4%; 60.7-66.0) [21] | 54/77 (70.1%) [1] | 127/300 (42.3%; 36.9-48.0) [2] |  | 181/377 (49.8%; 44.8-54.9) [3] |
| Fluoroquinolones | 68.0% (62.4-73.2) [5] | 79.0% (74.4-83.0) [4] | 0.0% [1] | 56.5% (51.4-61.6) [10] | 63.2% (60.5-65.9) [19] | 47.1% [1] | 63.0% (57.4-68.3) [2] | 3.3% [1] | 55.0% (50.1-59.9) [3] |
| Ciprofloxacin | 181/283 (68.0%; 62.4-73.2) [5] | 266/335 (79.0%; 74.4-83.0) [4] |  | 506/1322 (38.3%; 35.7-40.9) [18] | 953/1940 (50.3%; 48.1-52.6) [27] | 48/102 (47.1%) [1] | 189/300 (63.0%; 57.4-68.3) [2] | 82/2500 (3.3%) [1] | 319/2902 (11.1%; 10.0-12.3) [4] |
| Levofloxacin | 186/318 (59.5%; 54.0-64.7) [4] | 365/512 (71.0%; 66.9-74.8) [4] | 0/2 (0.0%) [1] | 203/359 (56.5%; 51.4-61.6) [10] | 754/1191 (63.2%; 60.5-65.9) [19] | 40/96 (41.7%) [1] | 180/300 (60.0%; 54.4-65.4) [2] |  | 220/396 (55.0%; 50.1-59.9) [3] |
| Moxifloxacin |  |  |  | 10/28 (36.2%; 21.1-54.6) [4] | 10/28 (36.2%; 21.1-54.6) [4] |  |  |  |  |
| Penicillins |  |  |  |  |  |  |  |  |  |
| Ampicillin | 177/196 (90.5%; 85.6-93.9) [2] | 519/547 (94.9%; 92.7-96.4) [6] | 5/5 (100.0%; 56.6-100.0) [2] | 1009/1357 (72.2%; 69.8-74.5) [14] | 1710/2105 (80.9%; 79.2-82.5) [24] | 12/14 (9.8%; 2.2-34.7) [2] | 248/299 (83.0%; 78.3-86.8) [2] | 849/2500 (34.0%) [1] | 1109/2813 (38.0%; 36.2-39.8) [5] |
| Polymyxin |  |  |  |  |  |  |  |  |  |
| Colistin |  |  |  | 0/4 (0.0%) [1] | 0/4 (0.0%) [1] |  |  |  |  |
| Sulfonamides |  |  |  |  |  |  |  |  |  |
| Co-trimoxazole | 133/191 (69.2%; 62.3-75.3) [3] | 322/430 (74.9%; 70.6-78.8) [6] | 2/3 (66.7%) [1] | 832/1438 (58.1%; 55.5-60.6) [15] | 1289/2062 (63.6%; 61.5-65.6) [25] | 30/47 (63.8%) [1] | 168/307 (54.7%; 49.1-60.2) [3] | 715/2500 (28.6%) [1] | 913/2854 (32.7%; 31.0-34.4) [5] |
| Other antibiotics |  |  |  |  |  |  |  |  |  |
| Fosfomycin | 25/108 (23.1%) [1] | 37/511 (7.4%; 5.4-10.0) [4] | 1/3 (33.3%) [1] | 92/516 (29.8%; 26.0-33.9) [8] | 155/1138 (16.0%; 14.0-18.2) [14] |  | 6/294 (2.0%; 0.9-4.4) [2] |  | 6/294 (2.0%; 0.9-4.4) [2] |
| Nitrofurantoin |  | 89/368 (24.1%; 20.0-28.7) [2] |  | 36/262 (17.0%; 12.9-22.0) [2] | 125/630 (23.3%; 20.2-26.8) [4] |  | 38/280 (13.6%) [1] |  | 38/280 (13.6%) [1] |

The data are presented as: number of resistant isolates (numerator)/number of total isolates (denominator) (AMR prevalence estimates; 95%CI) [N studies]. 95% confidence intervals were estimated using Wilson score interval where the number of studies was greater than one.

# Table S9. Numbers and proportions of resistant isolates for *Pseudomonas aeruginosa*-antimicrobial combinations

| Antibiotics | Hospitals | | | | Communities | | |
| --- | --- | --- | --- | --- | --- | --- | --- |
|  | **Blood** | **Lower respiratory tract** | **Other** | **Total** | **Blood** | **Lower respiratory tract** | **Total** |
| Aminoglycosides |  |  |  |  |  |  |  |
| Amikacin | 130/500 (26.2%; 22.5-30.2) [8] | 43/104 (41.3%; 32.4-51.0) [5] | 146/434 (33.6%; 29.4-38.2) [15] | 319/1038 (30.8%; 28.1-33.7) [25] | 1/54 (1.9%) [1] |  | 1/54 (1.9%) [1] |
| Gentamicin | 217/498 (43.9%; 39.6-48.3) [8] | 49/104 (47.1%; 37.8-56.6) [5] | 215/428 (50.2%; 45.5-54.9) [17] | 481/1030 (46.9%; 43.8-49.9) [27] | 6/49 (12.2%) [1] | 0/8 (0.0%) [1] | 6/57 (10.8%; 5.1-21.5) [2] |
| Tobramycin | 88/199 (44.6%; 37.8-51.5) [4] | 24/39 (60.1%; 44.5-73.9) [2] | 90/125 (72.0%; 63.6-79.1) [3] | 202/363 (56.0%; 50.9-61.0) [6] | 7/57 (12.3%) [1] |  | 7/57 (12.3%) [1] |
| Carbapenems | 24.0% (17.9-31.4) [3] | 70.7% (55.5-82.4) [2] | 72.7% [1] | 35.8% (33.1-38.6) [24] | 10.2% [1] | 0.0% [1] | 9.0% (3.9-19.2) [2] |
| Doripenem | 36/151 (24.0%; 17.9-31.4) [3] |  | 8/11 (72.7%) [1] | 44/162 (26.4%; 20.2-33.6) [4] | 3/36 (8.3%) [1] |  | 3/36 (8.3%) [1] |
| Imipenem | 79/364 (21.3%; 17.4-25.8) [7] | 29/41 (70.7%; 55.5-82.4) [2] | 264/623 (42.3%; 38.5-46.2) [14] | 372/1028 (35.4%; 32.5-38.4) [20] | 4/45 (8.9%) [1] |  | 4/45 (8.9%) [1] |
| Meropenem | 65/292 (22.8%; 18.4-28.0) [5] | 50/86 (58.3%; 47.8-68.2) [5] | 292/758 (38.5%; 35.1-42.0) [15] | 407/1136 (35.8%; 33.1-38.6) [24] | 5/49 (10.2%) [1] | 0/8 (0.0%) [1] | 5/57 (9.0%; 3.9-19.2) [2] |
| Third generation  cephalosporins | 22.5% (19.0-26.5) [8] | 50.0% (40.6-59.4) [5] | 46.3% (41.4-51.3) [13] | 34.8% (31.8-37.8) [23] | 10.0% [1] | 0.0% [1] | 8.8% (3.6-20.2) [2] |
| Ceftazidime | 106/475 (22.5%; 19.0-26.5) [8] | 52/104 (50.0%; 40.6-59.4) [5] | 182/393 (46.3%; 41.4-51.3) [13] | 340/972 (34.8%; 31.8-37.8) [23] | 4/40 (10.0%) [1] | 0/8 (0.0%) [1] | 4/48 (8.8%; 3.6-20.2) [2] |
| Fluoroquinolones | 22.4% (18.6-26.7) [7] | 59.0% (48.3-69.0) [4] | 59.5% (52.9-65.8) [9] | 43.9% (39.5-48.4) [15] | 2.2% [1] | 50.0% [1] | 7.8% (3.1-18.1) [2] |
| Ciprofloxacin | 89/402 (22.4%; 18.6-26.7) [7] | 49/104 (47.1%; 37.8-56.6) [5] | 216/421 (51.3%; 46.5-56.0) [15] | 354/927 (37.7%; 34.7-40.9) [24] | 1/46 (2.2%) [1] | 4/8 (50.0%) [1] | 5/54 (7.8%; 3.1-18.1) [2] |
| Levofloxacin | 31/177 (17.5%; 12.6-23.8) [3] | 49/83 (59.0%; 48.3-69.0) [4] | 130/218 (59.5%; 52.9-65.8) [9] | 210/478 (43.9%; 39.5-48.4) [15] |  |  |  |
| Penicillins |  |  |  |  |  |  |  |
| Piperacillin/Tazobactam | 12/56 (21.4%) [1] | 43/77 (55.8%; 44.7-66.4) [3] | 102/211 (48.3%; 41.7-55.1) [8] | 157/344 (45.6%; 40.5-50.9) [11] |  |  |  |
| Polymyxin |  |  |  |  |  |  |  |
| Colistin |  | 41/41 (100.0%) [1] | 1/1 (100.0%) [1] | 42/42 (100.0%; 91.6-100.0) [2] |  |  |  |

The data are presented as: number of resistant isolates (numerator)/ number of total isolates (denominator) (AMR prevalence estimates; 95%CI) [N studies]. 95% confidence intervals were estimated using Wilson score interval where the number of studies was greater than one.

# Table S10. Numbers and proportions of resistant isolates for *Staphylococcus aureus*-antimicrobial combinations

| Antibiotics | Hospitals | | | | | Communities | | | |
| --- | --- | --- | --- | --- | --- | --- | --- | --- | --- |
|  | **Blood** | **Cerebrospinal fluid** | **Lower respiratory tract** | **Other** | **Total** | **Blood** | **Lower respiratory tract** | **Other** | **Total** |
| Macrolides |  |  |  |  |  |  |  |  |  |
| Erythromycin | 15/74 (20.3%) [1] |  | 2/5 (40.0%) [1] | 105/348 (30.2%; 25.6-35.2) [8] | 122/427 (28.6%; 24.5-33.0) [10] |  | 0/11 (0.0%) [1] | 14/306 (10.6%; 7.7-14.6) [2] | 14/317 (10.4%; 7.5-14.3) [3] |
| Penicillins |  |  |  |  |  |  |  |  |  |
| Methicillin^a^ | 34/157 (21.5%; 15.8-28.6) [2] | 2/5 (40.0%) [1] | 32/97 (33.3%; 24.7-43.1) [3] | 120/572 (20.4%; 17.3-23.9) [11] | 188/831 (22.2%; 19.5-25.1) [14] |  |  | 9/367 (11.1%; 8.3-14.7) [3] | 9/367 (11.1%; 8.3-14.7) [3] |
| Other antibiotics |  |  |  |  |  |  |  |  |  |
| Clindamycin | 49/316 (17.3%; 13.5-21.8) [3] |  | 3/7 (42.9%; 15.8-75.0) [2] | 11/40 (47.5%; 32.9-62.5) [5] | 63/363 (22.0%; 18.0-26.5) [10] | 8/77 (13.4%; 7.5-22.8) [2] | 0/11 (0.0%) [1] | 2/62 (3.2%) [1] | 10/150 (9.6%; 5.9-15.4) [4] |
| Vancomycin | 35/613 (5.6%; 4.1-7.8) [4] |  | 5/334 (1.5%; 0.6-3.5) [3] | 17/739 (6.0%; 4.5-7.9) [12] | 57/1686 (5.0%; 4.1-6.1) [17] | 4/123 (3.2%; 1.3-8.0) [2] |  | 0/62 (0.0%) [1] | 4/185 (2.2%; 0.9-5.5) [3] |

The data are presented as: number of resistant isolates (numerator)/ number of total isolates (denominator) (AMR prevalence estimates; 95%CI) [N studies]. 95% confidence intervals were estimated using Wilson score interval where the number of studies was greater than one. ^a^Resistance to methicillin detected using either cefoxitin or oxacillin.

# Table S11. Numbers and proportions of resistant isolates for *Acinetobacter baumannii*-antimicrobial combinations

| Antibiotics | Hospitals | | | | Communities | | |
| --- | --- | --- | --- | --- | --- | --- | --- |
|  | **Blood** | **Lower respiratory tract** | **Other** | **Total** | **Blood** | **Lower respiratory tract** | **Total** |
| Aminoglycosides |  |  |  |  |  |  |  |
| Amikacin | 168/382 (44.0%; 39.1-49.0) [5] | 27/69 (39.1%; 28.5-50.9) [3] | 496/1780 (27.9%; 25.8-30.0) [16] | 691/2231 (31.0%; 29.1-32.9) [22] | 13/47 (27.7%) [1] |  | 13/47 (27.7%) [1] |
| Gentamicin | 295/382 (77.2%; 72.8-81.1) [5] | 50/69 (72.5%; 61.0-81.6) [3] | 1148/1747 (65.7%; 63.5-67.9) [16] | 1493/2198 (67.9%; 65.9-69.8) [22] | 11/42 (26.2%) [1] | 0/4 (0.0%) [1] | 11/46 (24.4%; 14.3-38.4) [2] |
| Carbapenems | 51.4% (45.3-57.5) [2] | 76.8% (65.6-85.2) [3] | 85.3% (81.8-88.3) [9] | 70.7% (67.4-73.8) [12] | 23.5% [1] | 0.0% [1] | 23.5% [1] |
| Doripenem | 125/252 (51.4%; 45.3-57.5) [2] |  | 17/22 (77.3%; 56.6-89.9) [2] | 142/274 (53.1%; 47.1-58.9) [4] | 8/34 (23.5%) [1] |  | 8/34 (23.5%) [1] |
| Imipenem | 159/316 (50.3%; 44.8-55.8) [4] |  | 387/453 (85.3%; 81.8-88.3) [9] | 546/769 (70.7%; 67.4-73.8) [12] | 6/36 (16.7%) [1] |  | 6/36 (16.7%) [1] |
| Meropenem | 178/378 (47.1%; 42.1-52.2) [5] | 53/69 (76.8%; 65.6-85.2) [3] | 1270/2103 (60.4%; 58.3-62.5) [18] | 1501/2550 (58.9%; 56.9-60.8) [24] | 9/47 (19.1%) [1] | 0/4 (0.0%) [1] | 9/51 (17.8%; 9.7-30.4) [2] |
| Tetracycline |  |  |  |  |  |  |  |
| Tigecycline | 37/211 (17.5%; 13.0-23.2) [3] | 30/69 (43.5%; 32.4-55.2) [3] | 479/1523 (31.5%; 29.2-33.9) [11] | 546/1803 (30.4%; 28.3-32.5) [16] | 9/47 (19.1%) [1] |  | 9/47 (19.1%) [1] |
| Polymyxin |  |  |  |  |  |  |  |
| Colistin |  |  | 47/99 (47.5%; 37.9-57.2) [4] | 47/99 (47.5%; 37.9-57.2) [4] |  |  |  |

The data are presented as: number of resistant isolates (numerator)/ number of total isolates (denominator) (AMR prevalence estimates; 95%CI) [N studies]. 95% confidence intervals were estimated using Wilson score interval where the number of studies was greater than one.

# Table S12. Numbers and proportions of resistant isolates for *Streptococcus pneumoniae*-antimicrobial combinations

| Antibiotics | Hospitals | | | | Communities | | |
| --- | --- | --- | --- | --- | --- | --- | --- |
|  | **Blood** | **Lower respiratory tract** | **Other** | **Total** | **Lower respiratory tract** | **Other** | **Total** |
| Carbapenems |  | 0.0% [1] | 13.5% [1] | 13.5% [1] |  |  |  |
| Imipenem |  |  | 5/37 (13.5%) [1] | 5/37 (13.5%) [1] |  |  |  |
| Meropenem |  | 0/14 (0.0%) [1] | 2/37 (5.4%) [1] | 2/51 (3.9%; 1.1-13.2) [2] |  |  |  |
| Third-generation  cephalosporins | 50.0% [1] | 7.1% [1] | 23.1% (12.6-38.3) [2] | 16.4% (8.9-28.3) [4] |  |  |  |
| Ceftriaxone | 1/2 (50.0%) [1] | 1/14 (7.1%) [1] | 7/94 (7.4%; 3.7-14.6) [3] | 9/110 (8.2%; 4.4-14.8) [5] |  |  |  |
| Cefotaxime | 0/2 (0.0%) [1] | 0/14 (0.0%) [1] | 9/39 (23.1%; 12.6-38.3) [2] | 9/55 (16.4%; 8.9-28.3) [4] |  |  |  |
| Macrolides |  |  |  |  |  |  |  |
| Erythromycin |  | 3/27 (11.1%; 3.9-28.1) [2] | 11/39 (28.2%; 16.5-43.8) [2] | 14/66 (21.2%; 13.1-32.5) [4] | 0/3 (0.0%) [1] | 80/513 (15.6%; 12.7-19.0) [8] | 80/516 (15.5%; 12.6-18.9) [9] |
| Penicillins |  |  |  |  |  |  |  |
| Oxacillin |  | 1/13 (7.7%) [1] | 16/37 (43.2%) [1] | 17/50 (34.0%; 22.4-47.8) [2] |  | 167/367 (45.5%; 40.5-50.6) [6] | 167/367 (45.5%; 40.5-50.6) [6] |
| Penicillin G | 2/3 (66.7%) [1] | 1/27 (3.7%; 0.7-18.3) [2] | 23/57 (40.4%; 28.6-53.3) [2] | 26/87 (29.9%; 21.3-40.2) [5] |  | 34/142 (23.9%) [1] | 34/142 (23.9%) [1] |
| Sulfonamides |  |  |  |  |  |  |  |
| Co-trimoxazole | 1/1 (100.0%) [1] | 7/27 (25.9%; 13.2-44.7) [2] | 32/74 (43.2%; 32.6-54.6) [2] | 40/102 (39.8%; 30.8-49.5) [5] |  | 320/633 (50.6%; 46.7-54.4) [9] | 320/633 (50.6%; 46.7-54.4) [9] |

The data are presented as: number of resistant isolates (numerator)/ number of total isolates (denominator) (AMR prevalence estimates; 95%CI) [N studies]. 95% confidence intervals were estimated using Wilson score interval where the number of studies was greater than one.

# Table S13. Numbers and proportions of resistant isolates for *Salmonella enterica* serovar Typhi and Paratyphi A (typhoidal)-antimicrobial combinations

| Antibiotics | Hospitals | | | Communities | |
| --- | --- | --- | --- | --- | --- |
|  | **Blood** | **Other** | **Total** | **Blood** | **Total** |
| Third-generation cephalosporins | 4.7% (2.6-8.2) [3] | 11.8% [1] | 4.7% (2.6-8.2) [3] | 0.0% [1] | 0.0% [1] |
| Ceftriaxone | 6/247 (2.5%; 1.2-5.3) [3] | 2/17 (11.8%) [1] | 8/264 (3.1%; 1.6-5.9) [4] | 0/262 (0.0%) [1] | 0/262 (0.0%) [1] |
| Ceftazidime | 0/50 (0.0%) [1] |  | 0/50 (0.0%) [1] |  |  |
| Cefotaxime | 11/230 (4.7%; 2.6-8.2) [3] |  | 11/230 (4.7%; 2.6-8.2) [3] |  |  |
| Fluoroquinolones | 2.1% (0.9-4.9) [3] | 13.0% (6.1-25.7) [3] | 2.8% (1.4-5.4) [6] | 0.0% [1] | 0.0% [1] |
| Ciprofloxacin | 2/241 (0.9%; 0.3-3.1) [3] | 6/46 (13.0%; 6.1-25.7) [3] | 8/287 (2.8%; 1.4-5.4) [6] | 0/262 (0.0%) [1] | 0/262 (0.0%) [1] |
| Levofloxacin | 5/239 (2.1%; 0.9-4.9) [3] | 1/45 (2.2%; 0.4-11.6) [2] | 6/284 (2.1%; 1.0-4.6) [5] |  |  |
| Moxifloxacin |  | 0/28 (0.0%) [1] | 0/28 (0.0%) [1] |  |  |
| Penicillins |  |  |  |  |  |
| Ampicillin | 10/168 (6.0%) [1] | 3/17 (17.6%) [1] | 13/185 (7.0%; 4.2-11.7) [2] | 2/262 (0.8%) [1] | 2/262 (0.8%) [1] |
| Sulfonamides |  |  |  |  |  |
| Co-trimoxazole | 14/168 (8.3%) [1] | 9/18 (50.0%; 29.0-71.0) [2] | 23/186 (12.4%; 8.4-17.9) [3] | 0/262 (0.0%) [1] | 0/262 (0.0%) [1] |
| Other antibiotics |  |  |  |  |  |
| Chloramphenicol |  | 3/17 (17.6%) [1] | 3/17 (17.6%) [1] | 2/262 (0.8%) [1] | 2/262 (0.8%) [1] |

The data are presented as: number of resistant isolates (numerator)/ number of total isolates (denominator) (AMR prevalence estimates; 95%CI) [N studies]. 95% confidence intervals were estimated using Wilson score interval where the number of studies was greater than one.

# Table S14. Numbers and proportions of resistant isolates for *Neisseria gonorrhoeae*-antimicrobial combinations

| Antibiotics | Communities | |
| --- | --- | --- |
|  | **Urethral, cervical, pharyngeal and rectal swabs** | **Total** |
| Aminoglycosides |  |  |
| Gentamicin | 1/163 (0.6%) [1] | 1/163 (0.6%) [1] |
| Third-generation cephalosporins | 3.5% (2.3-5.2) [6] | 3.5% (2.3-5.2) [6] |
| Ceftriaxone | 22/629 (3.5%; 2.3-5.2) [6] | 22/629 (3.5%; 2.3-5.2) [6] |
| Cefixime | 3/440 (0.7%; 0.2-2.0) [4] | 3/440 (0.7%; 0.2-2.0) [4] |
| Fluoroquinolones | 58.4% (54.4-62.3) [5] | 58.4% (54.4-62.3) [5] |
| Ciprofloxacin | 347/594 (58.4%; 54.4-62.3) [5] | 347/594 (58.4%; 54.4-62.3) [5] |
| Macrolides |  |  |
| Azithromycin | 6/568 (1.1%; 0.5-2.3) [4] | 6/568 (1.1%; 0.5-2.3) [4] |
| Penicillins |  |  |
| Penicillin G | 160/163 (98.2%) [1] | 160/163 (98.2%) [1] |
| Other antibiotics |  |  |
| Spectinomycin | 1/489 (0.2%; 0.0-1.1) [3] | 1/489 (0.2%; 0.0-1.1) [3] |

The data are presented as: number of resistant isolates (numerator)/ number of total isolates (denominator) (AMR prevalence estimates; 95%CI) [N studies]. 95% confidence intervals were estimated using Wilson score interval where the number of studies was greater than one.

# Table S15. Numbers and proportions of resistant isolates for *Shigella spp*.-antimicrobial combinations

| Antibiotics | Hospitals | | | Communities | |
| --- | --- | --- | --- | --- | --- |
|  | **Stool^a^** | **Other** | **Total** | **Stool^a^** | **Total** |
| Third-generation cephalosporins | 0.0% (0.0-0.4) [3] | 5.1% [1] | 5.1% [1] | 0.0% [1] | 0.0% [1] |
| Ceftriaxone | 0/1068 (0.0%; 0.0-0.4) [3] | 1/39 (2.6%) [1] | 1/1107 (0.1%; 0.0-0.5) [4] | 0/57 (0.0%) [1] | 0/57 (0.0%) [1] |
| Cefotaxime |  | 2/39 (5.1%) [1] | 2/39 (5.1%) [1] |  |  |
| Fluoroquinolones | 0.0% (0.0-0.4) [3] | 10.3% [1] | 0.4% (0.1-0.9) [4] | 0.0% [1] | 0.0% [1] |
| Ciprofloxacin | 0/1068 (0.0%; 0.0-0.4) [3] | 4/39 (10.3%) [1] | 4/1107 (0.4%; 0.1-0.9) [4] | 0/57 (0.0%) [1] | 0/57 (0.0%) [1] |
| Macrolides |  |  |  |  |  |
| Azithromycin |  |  |  | 2/57 (3.5%) [1] | 2/57 (3.5%) [1] |
| Sulfonamides |  |  |  |  |  |
| Co-trimoxazole | 732/1068 (68.5%; 65.7-71.3) [3] |  | 732/1068 (68.5%; 65.7-71.3) [3] | 40/57 (70.2%) [1] | 40/57 (70.2%) [1] |

The data are presented as: number of resistant isolates (numerator)/ number of total isolates (denominator) (AMR prevalence estimates; 95%CI) [N studies]. 95% confidence intervals were estimated using Wilson score interval where the number of studies was greater than one. ^a^Stool specimens include rectal swabs.

# Table S16. Numbers and proportions of resistant isolates for *Haemophilus influenzae*-antimicrobial combinations

| Antibiotics | Hospitals | | Communities | |
| --- | --- | --- | --- | --- |
|  | **Other** | **Total** | **Other** | **Total** |
| Third-generation cephalosporins | 2.9% [1] | 2.9% [1] | 0.0% [1] | 0.0% [1] |
| Ceftriaxone |  |  | 0/85 (0.0%) [1] | 0/85 (0.0%) [1] |
| Cefotaxime | 1/34 (2.9%) [1] | 1/34 (2.9%) [1] |  |  |
| Fluoroquinolones | 3.7% (1.5-9.2) [2] | 3.7% (1.5-9.2) [2] | 0.0% [1] | 0.0% [1] |
| Levofloxacin | 4/107 (3.7%; 1.5-9.2) [2] | 4/107 (3.7%; 1.5-9.2) [2] | 0/85 (0.0%) [1] | 0/85 (0.0%) [1] |
| Penicillins |  |  |  |  |
| Amoxicillin-clavulanic acid |  |  | 12/85 (14.1%) [1] | 12/85 (14.1%) [1] |
| Ampicillin | 13/34 (38.2%) [1] | 13/34 (38.2%) [1] | 1/1 (100.0%) [1] | 1/1 (100.0%) [1] |
| Sulfonamides |  |  |  |  |
| Co-trimoxazole | 20/34 (58.8%) [1] | 20/34 (58.8%) [1] | 22/85 (25.9%) [1] | 22/85 (25.9%) [1] |

The data are presented as: number of resistant isolates (numerator)/ number of total isolates (denominator) (AMR prevalence estimates; 95%CI) [N studies]. 95% confidence intervals were estimated using Wilson score interval where the number of studies was greater than one.

# Table S17. Numbers and proportions of resistant isolates for *Salmonella spp.* (non-typhoidal)-antimicrobial combinations

| Antibiotics | Hospitals | | | Communities | | |
| --- | --- | --- | --- | --- | --- | --- |
|  | **Blood** | **Stool^a^** | **Total** | **Blood** | **Stool^a^** | **Total** |
| Carbapenems | 16.7% [1] |  | 16.7% [1] | 5.3% [1] |  | 5.3% [1] |
| Doripenem | 1/16 (6.3%) [1] |  | 1/16 (6.3%) [1] | 0/19 (0.0%) [1] |  | 0/19 (0.0%) [1] |
| Imipenem | 2/12 (16.7%) [1] |  | 2/12 (16.7%) [1] | 1/19 (5.3%) [1] |  | 1/19 (5.3%) [1] |
| Meropenem | 1/16 (6.3%) [1] |  | 1/16 (6.3%) [1] | 0/24 (0.0%) [1] |  | 0/24 (0.0%) [1] |
| Third-generation cephalosporins | 26.7% [1] | 0.0% [1] | 26.7% [1] | 10.5% [1] | 100.0% [1] | 70.6% (59.6-79.6) [2] |
| Ceftriaxone | 2/13 (15.4%) [1] | 0/158 (0.0%) [1] | 2/171 (1.6%; 0.5-4.8) [2] | 2/21 (9.5%) [1] | 56/56 (100.0%) [1] | 58/77 (70.6%; 59.6-79.6) [2] |
| Ceftazidime | 3/13 (23.1%) [1] |  | 3/13 (23.1%) [1] | 2/20 (10.0%) [1] |  | 2/20 (10.0%) [1] |
| Cefotaxime | 4/15 (26.7%) [1] |  | 4/15 (26.7%) [1] | 2/19 (10.5%) [1] |  | 2/19 (10.5%) [1] |
| Fluoroquinolones | 16.7% [1] | 1.9% [1] | 16.7% [1] | 11.1% [1] | 100.0% [1] | 71.1% (60.6-79.7) [2] |
| Ciprofloxacin | 3/18 (16.7%) [1] | 3/158 (1.9%) [1] | 6/176 (3.4%; 1.6-7.2) [2] | 3/27 (11.1%) [1] | 56/56 (100.0%) [1] | 59/83 (71.1%; 60.6-79.7) [2] |
| Levofloxacin | 3/18 (16.7%) [1] |  | 3/18 (16.7%) [1] | 3/27 (11.1%) [1] |  | 3/27 (11.1%) [1] |
| Sulfonamides |  |  |  |  |  |  |
| Co-trimoxazole |  | 13/158 (8.2%) [1] | 13/158 (8.2%) [1] |  | 5/56 (8.9%) [1] | 5/56 (8.9%) [1] |

The data are presented as: number of resistant isolates (numerator)/ number of total isolates (denominator) (AMR prevalence estimates; 95%CI) [N studies]. 95% confidence intervals were estimated using Wilson score interval where the number of studies was greater than one. ^a^Stool specimens include rectal swabs.

# References

1 Adhima F, Wahyunitisari MR, Prasetyo RV, Setiabudi RJ. Bacterial Profile and Antibiotic Resistance Pattern among Children with Urinary Tract Infections in Dr. Soetomo Hospital, Surabaya, Indonesia. *Indones J Trop Infect Dis* 2022; : 123–36.

2 Afifurrahman, Samadin KH, Aziz S. Pola Kepekaan Bakteri Staphylococcus aureus terhadap Antibiotik Vancomycin di RSUP Dr . Mohammad Hoesin Palembang. *Maj Kedokt Sriwij* 2014; **46**: 266–70.

3 Akbar GT, Karimi J, Anggraini D. Pola bakteri dan resistensi antibiotik pada ulkus diabetik grade dua di RSUD Arifin Achmad periode 2012. *J Online Mhs Fak Kedokt* 2014; **2**: 1–15.

4 Amanda G, Tafroji W, Sutoyo DK, Burhan E, Haryanto B, Safari D. Serotype distribution and antimicrobial profile of Streptococcus pneumoniae isolated from adult patients with community-acquired pneumonia in Jakarta, Indonesia. *J Microbiol Immunol Infect* 2021; **54**: 1175–8.

5 Anggraini D, Kemal RA, Hadi U, Kuntaman K. The susceptibility pattern and distribution of blaOXA-23 genes of clinical isolate Acinetobacter baumannii in a tertiary hospital, Indonesia. *J Infect Dev Ctries* 2022; **16**: 821–6.

6 Ariana N, Pestariati P, Sasongkowati R, Kusumaningrum D. Resistance Pattern of Escherichia Coli Againts Antibiotics In Urinary Tract Infection Patients In RSUD Dr Soetomo Surabaya. *J Community Med Public Heal Res* 2020; **1**: 53–8.

7 Artono, Surayya R, Purnami N, Handoko E. Microbiological profile with Antibiotic Sensitivity Pattern for Chronic Suppurative Otitis Media in A Tertiary Hospital, Indonesia. *Res J Pharm Technol* 2022; **15**: 1683–8.

8 Azwar A, Salawati L. Isolasi, Identifikasi Dan Uji Resistensi Antibiotika Mikroorganisme dari Sputum Penderita Batuk Kronis. *J Kedokt Syiah Kuala* 2012; **12**.

9 Bukitwetan P, Suryawidjaja JE, Salim OC, Hidayat A, Herwana E, Lesmana M. Serovar distribution and antibiotic susceptibility of nontyphoidal Salmonella isolated from pediatric patients in Jakarta, Indonesia. *Southeast Asian J Trop Med Public Health* 2007; **38**: 1088–94.

10 Chudlori B, Kuswandi M, Indrayudha P. Pola kuman dan resistensinya terhadap antibiotika dari spesimen pus di RSUD Dr. Moewardi tahun 2012. *Pharmacon* 2012; **13**: 70–6.

11 Chung DR, Song JH, Kim SH, *et al.* High prevalence of multidrug-resistant nonfermenters in hospital-acquired pneumonia in Asia. *Am J Respir Crit Care Med* 2011; **184**: 1409–17.

12 Deurenberg RH, Beisser PS, Visschers MJ, Driessen C, Stobberingh EE. Molecular typing of methicillin-susceptible Staphylococcus aureus isolates collected in the Yogyakarta area in Indonesia, 2006. *Clin Microbiol Infect* 2010; **16**: 92–4.

13 Devian MK, Suranadi IW, Hartawan IGAGU, Aryabiantara IW. Bacterial Patterns and Sensitivity to Antibiotics in Patients Treated with Ventilators at the Intensive Care Unit of Sanglah Hospital Denpasar, Bali, Indonesia. *Open Access Maced J Med Sci* 2022; **10**: 250–4.

14 Donegan EA, Wirawan DN, Muliawan P, *et al.* Fluoroquinolone-resistant Neisseria gonorrhoeae in Bali, Indonesia: 2004. *Sex Transm Dis* 2006; **33**: 625–9.

15 Dwipoerwantoro P, Pulungsih S, Susanti N, Sadikin H, Firmansyah A. A study on the antibiotic resistance of Shigella. *Paediatr Indones* 2016; **45**. DOI:10.14238/pi45.2.2005.49-54.

16 Fabian P, Alimsardjono L, Indiastuti DN. Pola resistensi bakteri Pseudomonas aeruginosa dan Acinetobacter baumannii pada spesimen darah terhadap antibiotik golongan β-laktam dan aminoglikosida di Rumah Sakit DR. Soetomo periode Januari 2016 – Desember 2016. *J Kedokt Syiah Kuala* 2020; **20**. DOI:10.24815/JKS.V20I1.18296.

17 Farida H, Severin JA, Gasem MH, *et al.* Nasopharyngeal carriage of Klebsiella pneumoniae and other Gram-negative bacilli in pneumonia-prone age groups in Semarang, Indonesia. *J Clin Microbiol* 2013; **51**: 1614–6.

18 Farida H, Severin JA, Gasem MH, *et al.* Nasopharyngeal carriage of Streptococcus pneumonia in pneumonia-prone age groups in Semarang, Java Island, Indonesia. *PLoS One* 2014; **9**: e87431.

19 Fathin A, Kusumawati L. Pola Resistensi Antibiotik Pada Pasien Dewasa yang Menderita Pneumonia di RS Usu Periode Januari 2017 – Desember 2018. *J Syntax Fusion J Nas Indones* 2022; **2**.

20 Firdiana SE, Muslimin M, Farida H. Perbandingan Efektivitas Seftriakson Dengan Siprofloksasin Pada Kuman Neisseria Gonorrhoeae Secara In Vitro. *J Kedokt Diponegoro (Diponegoro Med Journal)* 2016; **5**: 1736–42.

21 Hananta IPY, van Dam AP, Bruisten SM, Schim van der Loeff MF, Soebono H, de Vries HJC. Gonorrhea in Indonesia: High Prevalence of Asymptomatic Urogenital Gonorrhea but No Circulating Extended Spectrum Cephalosporins-Resistant Neisseria gonorrhoeae Strains in Jakarta, Yogyakarta, and Denpasar, Indonesia. *Sex Transm Dis* 2016; **43**: 608–16.

22 Harimurti K, Saldi SRF, Dewiasty E, *et al.* Nasopharyngeal carriage of Streptococcus pneumoniae in adults infected with human immunodeficiency virus in Jakarta, Indonesia. *J Infect Public Health* 2016; **9**: 633–8.

23 Harimurti K, Saldi SRF, Dewiasty E, *et al.* Streptococcus pneumoniae carriage and antibiotic susceptibility among Indonesian pilgrims during the Hajj pilgrimage in 2015. *PLoS One* 2021; **16**. DOI:10.1371/journal.pone.0246122.

24 Harnarnik S, Mulyaningsih S, Triastuti A. Uji Kepekaan Bakteri Escherichia coli Hasil Isolasi Dari Urin Pasien Rumah Sakit Dr. Sardjito Terhadap Antibiotic Golongan B-Laktam. *J Ilm Farm* 2005; **2**: 37–43.

25 Herdiyanti H, Indiastuti DN. Resistance Patterns of Escherichia coli and Klebsiella pneumoniae Bacteria Against Amikacin, Ceftazidime, Meropenem, Nitrofurantoin Antibiotics in Elderly Patients with UTI in RSUD Dr. Soetomo. *J Ilm Mhs Kedokt Univ Airlangga* 2019; **10**.

26 Herwana E, Surjawidjaja JE, Salim OC, Indriani N, Bukitwetan P, Lesmana M. Shigella-associated diarrhea in children in South Jakarta, Indonesia. *Southeast Asian J Trop Med Public Health* 2010; **41**: 418–25.

27 Hidayah AN, Hasmono D, Thayyib M, Kuntaman K. The Pattern of Antibiotic Prescription and Antimicrobial Resistance of Gut Flora Escherichia coli at Aisyiyah Hospital, Bojonegoro. *Folia Medica Indones* 2020; **56**: 99.

28 Indraningrat AAG, Masyeni DAPS, Darmana IMS. Retrospective Analysis on Antibiotic Resistance among Clinical Bacterial Isolates in the General Hospital Sanjiwani, Gianyar Bali. *J Kesehat Indones* 2022; **12**: 159–64.

29 Indri SS, Hertanti IL, Rizka R. Pola Kepekaan Bakteri Penyebab Infeksi Saluran Kemih pada Anak terhadap Antimikroba. *Maj Kedokt Sriwij* 2015; **47**: 85–90.

30 Indriatmi W, Pragoyo RL, Nilasari H, Suseno LS. Antimicrobial resistance of Neisseria gonorrhoeae in Jakarta, Indonesia: a cross-sectional study. *Sex Health* 2020; **17**: 9–14.

31 Karmila A, Barchia I, Ramandati A, Zhang L. Clinical and bacteriological profile of culture-negative and culture-proven neonatal sepsis in Palembang, Indonesia. *J Infect Dev Ctries* 2022; **16**: 1887–96.

32 Kartasasmita CB, Duddy HM, Sunaryati S, *et al.* Nasopharyngeal bacterial carriage and antimicrobial resistance in underfive children with community acquired pneumonia. *Med J Indones* 2002; **11**: 164–9.

33 Khalilullah S, Susanti D, Saputra RW, Nurjannah. Trends antimicrobial resistance in urinary tract infections: a research at the Dr. Zainoel Abidin Teaching Hospital, Banda Aceh, Indonesia. *Proc Annu Int Conf Syiah Kuala Univ* 2011; **1**.

34 Kitagawa K, Shigemura K, Yamamichi F, *et al.* International Comparison of Causative Bacteria and Antimicrobial Susceptibilities of Urinary Tract Infections between Kobe, Japan, and Surabaya, Indonesia. *Jpn J Infect Dis* 2018; **71**: 8–13.

35 Lestari ES, Severin JA, Filius PMG, *et al.* Antimicrobial resistance among commensal isolates of Escherichia coli and Staphylococcus aureus in the Indonesian population inside and outside hospitals. *Eur J Clin Microbiol Infect Dis* 2008; **27**: 45–51.

36 Lestari ES, Duerink DO, Hadi U, *et al.* Determinants of carriage of resistant Staphylococcus aureus among S. aureus carriers in the Indonesian population inside and outside hospitals. *Trop Med Int Health* 2010; **15**: 1235–43.

37 Lestari PI, Susanti I, Rahmawati H. Pola Kepekaan Bakteri terhadap Antibiotik di Ruang Rawat Intensif RSPI Prof. Dr. Sulianti Saroso Jakarta. *Indones J Infect Dis* 2013; **1**: 23–7.

38 Lugito NPH, Cucunawangsih. Antimicrobial Resistance of Salmonella enterica Serovars Typhi and Paratyphi Isolates from a General Hospital in Karawaci, Tangerang, Indonesia: A Five-Year Review. *Int J Microbiol* 2017; **2017**: 6215136.

39 Mayasari A. Klebsiella Pneumoniae Identification Of Producing Extended Spectrum Beta Lactamase (ESBL) And Resistance Pattern Of Antibiotics In Ulin General Hospital Banjarmasin. *Berk Kedokt* 2017; **13**.

40 Meriyani H, Dwi AS, Sutariani NW, Juanita A, Siada NB. Antibiotic Use and Resistance at Intensive Care Unit of a Regional Public Hospital in Bali: A 3-Year Ecological Study. *J Farm Klin Indones* 2021; **10**: 180–9.

41 Meta DT, Endriani R, Sembiring LP. Identifikasi dan resistensi bakteri methicillin-resistant Staphylococcus aureus (MRSA) dari ulkus diabetikum derajat I dan II Wagner di bagian penyakit dalam RSUD Arifin Achmad. *J Online Mhs Fak Kedokt* 2014; **1**: 1–10.

42 Moehario LH, Tjoa E, Kiranasari A, *et al.* Trends in antimicrobial susceptibility of Gram-negative bacteria isolated from blood in Jakarta from 2002 to 2008. *J Infect Dev Ctries* 2009; **3**: 843–8.

43 Moehario LH, Esterita T, Shirleen V, Robertus T, Angelina Y. Association of acinetobacter baumannii with invasive procedures in hospitalized patients in jakarta. *J Infect Dev Ctries* 2020; **14**: 1455–60.

44 Muktiarti D, Khoeri MM, Tafroji W, Waslia L, Safari D. Serotypes and antibiotic susceptibility profile of Streptococcus pneumoniae isolated from nasopharynges of children infected with HIV in Jakarta, Indonesia, pre- and post-pneumococcal vaccination. *Access Microbiol* 2021; **3**. DOI:10.1099/ACMI.0.000215.

45 Murni IK, Duke T, Daley AJ, Kinney S, Soenarto Y. ANTIBIOTIC RESISTANCE AND MORTALITY IN CHILDREN WITH NOSOCOMIAL BLOODSTREAM INFECTION IN A TEACHING HOSPITAL IN INDONESIA. *Southeast Asian J Trop Med Public Health* 2016; **47**: 983–93.

46 Nathania I, Nainggolan IM, Yasmon A, *et al.* Hotspots sequences of gyrA, gyrB, parC, and parE genes encoded for fluoroquinolones resistance from local Salmonella Typhi strains in Jakarta. *BMC Microbiol* 2022; **22**: 1–13.

47 Nirwati H, Hakim MS, Darma S, Mustafa M, Nuryastuti T. Detection of blaoxa genes and identification of biofilm-producing capacity of Acinetobacter baumannii in a tertiary teaching hospital, Klaten, Indonesia. *Med J Malaysia* 2018; **73**: 291–6.

48 Nirwati H, Sinanjung K, Fahrunissa F, *et al.* Biofilm formation and antibiotic resistance of Klebsiella pneumoniae isolated from clinical samples in a tertiary care hospital, Klaten, Indonesia. *BMC Proc* 2019; **13**: 20.

49 Nur A, Marissa N. Description of diabetic ulcers bacteria at Zainal Abidin and Meuraxa Hospitals in 2015. *Bul Penelit Kesehat* 2016; **44**: 187–96.

50 Oyofo B, Lesmana M, Subekti D, *et al.* Surveillance of bacterial pathogens of diarrhea disease in Indonesia. *Diagn Microbiol Infect Dis* 2002; **44**: 227–34.

51 Parut AA. RESISTENSI ANTIBIOTIK PADA IBU HAMIL DENGAN BAKTERIURIA ASIMPTOMATIK (Antibiotic Resistance in Pregnant Women with Asymptomatic Bacteriuria). *J Ners Lentera* 2015; **3**: 51–7.

52 Patilaya P, Husori D, Marhafanny L. Susceptibility of Klebsiella Pneumoniae Isolated from Pus Specimens of Post-Surgery Patients in Medan, Indonesia to Selected Antibiotics. *Open Access Maced J Med Sci* 2019; **7**: 3861–4.

53 Pelegrin AC, Saharman YR, Griffon A, *et al.* Erratum for: High-risk international clones of carbapenem-nonsusceptible pseudomonas aeruginosa endemic to indonesian intensive care units: Impact of a multifaceted infection control intervention analyzed at the genomic level (mBio, (2019) 10, 6, 10.1128/. *MBio* 2020; **11**: 1.

54 Permana H, Saboe A, Soetedjo NN, Kartika D, Alisjahbana B. Empirical Antibiotic for Diabetic Foot Infection in Indonesian Tertiary Hospital, Is It Time to Rethink the Options? *Acta Med Indones* 2022; **54**: 247–54.

55 Prabowo F, Habib I. Identifikasi Pola Kepekaan dan Jenis Bakteri pada Pasien Infeksi Saluran Kemih di Rumah Sakit PKU Muhammadiyah Yogyakarta. *J Kedokt Dan Kesehat* 2012; **12**.

56 Punjabi NH, Agtini MD, Ochiai RL, *et al.* Enteric fever burden in North Jakarta, Indonesia: a prospective, community-based study. *J Infect Dev Ctries* 2013; **7**: 781–7.

57 Purba A, Ascobat P, Muchtar A, *et al.* Multidrug-Resistant Infections Among Hospitalized Adults With Community-Acquired Pneumonia In An Indonesian Tertiary Referral Hospital. *Infect Drug Resist* 2019; **12**: 3663–75.

58 Puspandari N, Sariadji K, Pangerti Yekti R, *et al.* Prevalensi dan Pola Resistensi N.gonorrhoeae Terhadap Beberapa Antibiotik pada Wanita Penjaja Seks di Jakarta Timur, Tangerang dan Palembang Tahun 2012. *J Biotek Medisiana Indones* 2016; **5**: 57–67.

59 Rachman NO, Prenggono MD, Budiarti LY. Uji Sensitivitas Bakteri Penyebab Infeksi Saluran Kemih Pada Pasien Diabetes Melitus Terhadap Seftriakson, Levofloksasin, Dan Gentamisin. *Berk Kedokt* 2016; **12**: 205.

60 Radji M, Fauziah S, Aribinuko N, M. R, S. F. Antibiotic sensitivity pattern of bacterial pathogens in the intensive care unit of Fatmawati Hospital, Indonesia. *Asian Pac J Trop Biomed* 2011; **1**: 39–42.

61 Radji M, Putri CS, Fauziyah S. Antibiotic therapy for diabetic foot infections in a tertiary care hospital in Jakarta, Indonesia. *Diabetes Metab Syndr Clin Res Rev* 2014; **8**: 221–4.

62 Rahman V, Anggraini D, Fauziah D. Pola Resistensi Acinetobacter Baumannii Yang Diisolasi Di Intensive Care Unit (Icu) Rsud Arifin Achmad Provinsi Riau Periode 1 Januari Hingga 31 Desember 2014. *J Online Mhs Fak Kedokt* 2015; **2**: 1–8.

63 Ramdhani D, Kusuma SAF, Azizah SN, Khumairoh I, Bap H, Sediana D. Profile of Antibiotic Resistance Against Influenza in Adult Patients: A Case Study at City Health Center in Indonesia. *Int J Appl Pharm* 2022; **14**: 101–4.

64 Rosana Y, Ocviyanti D, Halim M, *et al.* Urinary Tract Infections among Indonesian Pregnant Women and Its Susceptibility Pattern. *Infect Dis Obstet Gynecol* 2020; **2020**. DOI:10.1155/2020/9681632.

65 Rosana Y, Ocviyanti D, Akbar W. Bacterial susceptibility patterns to Co-trimoxazole in urinary tract infections of outpatients and inpatients in jakarta, indonesia. *Med J Indones* 2020; **29**: 316–21.

66 Rosyid AN, Endraswari PD, Kusmiati T, *et al.* The Spectrum of Cefditoren for Lower Respiratory Tract Infections (LR-TIs) in Surabaya. *Curr Drug ther* 2022; **17**: 30–8.

67 Safari D, Kurniati N, Waslia L, *et al.* Serotype distribution and antibiotic susceptibility of Streptococcus pneumoniae strains carried by children infected with human immunodeficiency virus. *PLoS One* 2014; **9**: e110526.

68 Safari D, Harimurti K, Khoeri MM, *et al.* Staphylococcus aureus and Streptococcus pneumoniae Prevalence Among Elderly Adults in Jakarta, Indonesia. *Southeast Asian J Trop Med Public Health* 2015; **46**: 465–71.

69 Safari D, Lestari AN, Khoeri MM, *et al.* Nasopharyngeal carriage and antimicrobial susceptibility profile of Haemophilus influenzae among patients infected with HIV in Jakarta, Indonesia. *Access Microbiol* 2020; **2**. DOI:10.1099/ACMI.0.000165.

70 Safari D, Widhidewi NW, Paramaiswari WT, *et al.* Prevalence, serotype distribution, and antimicrobial susceptibility profile of Streptococcus pneumoniae among patients with acute respiratory tract infection. *WHO South-East Asia J public Heal* 2022; **10**: 66–9.

71 Safari D, Wahyono DJ, Tafroji W, *et al.* Serotype Distribution and Antimicrobial Resistance Profile of Haemophilus influenzae Isolated from School Children with Acute Otitis Media. *Int J Microbiol* 2022; **2022**. DOI:10.1155/2022/5391291.

72 Sahara N, Hidayat H, Mauladi G, Sjahriani T. Dr. H. Abdul Moeloek Hospital Antibiotic Resistance Pattern. *J Kebidanan Malahayati* 2023; **9**: 1–14.

73 Saharman YR, Karuniawati A, Sedono R, *et al.* Endemic carbapenem-nonsusceptible Acinetobacter baumannii-calcoaceticus complex in intensive care units of the national referral hospital in Jakarta, Indonesia. *Antimicrob Resist Infect Control* 2018; **7**: 1–12.

74 Saharman YR, Pelegrin AC, Karuniawati A, *et al.* Epidemiology and characterisation of carbapenem-non-susceptible Pseudomonas aeruginosa in a large intensive care unit in Jakarta, Indonesia. *Int J Antimicrob Agents* 2019; **54**: 655–60.

75 Saharman YR, Karuniawati A, Sedono R, *et al.* Clinical impact of endemic NDM-producing Klebsiella pneumoniae in intensive care units of the national referral hospital in Jakarta, Indonesia. *Antimicrob Resist Infect Control* 2020; **9**: 1–14.

76 Said WF, Sukoto E, Khoeri MM, Kumalawati J, Safari D. Serotype distribution and antimicrobial susceptibility of Streptococcus pneumoniae isolates from adult patients in Jakarta, Indonesia. *J Infect Public Health* 2017; **10**: 833–5.

77 Salsabila K, Paramaiswari WT, Amalia H, *et al.* Nasopharyngeal carriage rate, serotype distribution, and antimicrobial susceptibility profile of Streptococcus pneumoniae isolated from children under five years old in Kotabaru, South Kalimantan, Indonesia. *J Microbiol Immunol Infect* 2021; **55**: 482–8.

78 Santoso P, Sung M, Hartantri Y, *et al.* MDR Pathogens Organisms as Risk Factor of Mortality in Secondary Pulmonary Bacterial Infections Among COVID-19 Patients: Observational Studies in Two Referral Hospitals in West Java, Indonesia. *Int J Gen Med* 2022; **15**: 4741–51.

79 Saputro A, Farida H, Firmanti SC. Perbedaan Pola Kepekaan Terhadap Antibiotik Pada Streptococcus pneumoniae Yang Mengkolonisasi Nasofaring Balita. *J Media Med Muda* 2013.

80 Sari, Iriani Y, Tjekyan RMS. Pola Kuman dan Resistensi Antibiotik di Pediatric Intensive Care Unit (PICU) RS. Dr. Mohammad Hoesin Palembang Tahun 2013. *J Kedokt dan Kesehat* 2015; **2**: 175–81.

81 Sari IP, Nuryastuti T, Wahyono D. The Study of Multidrug-Resistance in Neonatal Intensive Care Unit at the Central Java Hospital. *Asian J Pharm Clin Res* 2017; **10**: 80–4.

82 Setyati A, Murni IK. Pola Kuman Pasien Pneumonia di Instalasi Rawat Intensif Anak (IRIA) RSUP Dr. Sardjito. *Media Med Indones* 2012; **46**.

83 Sholeh MA, Kuntaman K, Hadi U. Quantity of Antibiotic Use and Resistance Pattern of Gut Normal Flora Escherichia coli at Intensive Care Unit and Tropic Infection Ward, Dr Soetomo Hospital, Surabaya, Indonesia. *Folia Medica Indones* 2020; **56**: 159–64.

84 Sinto R, Lie KC, Setiati S, *et al.* Blood culture utilization and epidemiology of antimicrobial-resistant bloodstream infections before and during the COVID-19 pandemic in the Indonesian national referral hospital. *Antimicrob Resist Infect Control* 2022; **11**: 73.

85 Soedarmono P, Diana A, Tauran P, *et al.* The characteristics of bacteremia among patients with acute febrile illness requiring hospitalization in Indonesia. *PLoS One* 2022; **17**: e0273414.

86 Subagdja M, Sugianli A, Prodjosoewojo S, Hartantri Y, Parwati I. Antibiotic Resistance in COVID-19 with Bacterial Infection: Laboratory-Based Surveillance Study at Single Tertiary Hospital in Indonesia. *Infect Drug Resist* 2022; **15**: 5849–56.

87 Sugianli AK, Ginting F, Kusumawati RL, *et al.* Antimicrobial resistance in uropathogens and appropriateness of empirical treatment: a population-based surveillance study in Indonesia. *J Antimicrob Chemother* 2017; **72**: 1469–77.

88 Suranadi IW, Panji PAS, Sri Budayanti NN, Senapathi TGA, Susatya AB. Evaluation of empirical meropenem bolus protocol in pseudomonas aeruginosa: A three-year analysis in tertiary intensive care unit. *Int J Gen Med* 2021; **14**: 7861–7.

89 Suryatenggara AN, Khoeri MM, Waslia L, *et al.* Identification and antibiotic susceptibility of methicillin-resistant Staphylococcus aureus strains collected at a referral hospital, Jakarta, Indonesia in 2013. *Southeast Asian J Trop Med Public Health* 2018; **49**: 1053–9.

90 Suryawati B, Saptawati L. A high incidence of multidrug resistant strains as the agents causing bloodstream infection in hospital in Indonesia. *Indones J Med* 2019; **4**: 28–34.

91 Sutrisna A, Soebjakto O, Wignall FS, *et al.* Increasing resistance to ciprofloxacin and other antibiotics in Neisseria gonorrhoeae from East Java and Papua, Indonesia, in 2004 - Implications for treatment. *Int J STD AIDS* 2006; **17**: 810–2.

92 Tan F, Triyono EA, Wahyunitisari MR. Microbial Patterns Of Hospitalized HIV Positive Patients Admitted In Dr. Soetomo General Hospital, Surabaya Indonesia. *J Vocat Heal Stud* 2021; **5**: 1–11.

93 Tandiono J, Marcella E, Pusung MK, Cucunawangsih C. Microorganism Spectrum and Its Sensitivity Pattern at Intensive Care Unit of a Secondary Care Teaching Hospital in Tangerang, Indonesia. *Open Access Maced J Med Sci* 2022; **10**: 761–6.

94 Tauran PM, Djaharuddin I, Bahrun U, *et al.* Excess mortality attributable to antimicrobial-resistant bacterial bloodstream infection at a tertiary-care hospital in Indonesia. *PLOS Glob Public Heal* 2022; **2**: e0000830.

95 Thirafi SZT, Sarassari R, Bramantono B, Kuntaman K. Susceptibility pattern of methicillin-resistant Staphylococcus aureus bacteria in Dr. Soetomo General Academic Hospital Surabaya. *J Berk Epidemiol / Period Epidemiol J* 2022; **10**: 331–40.

96 Tjaniadi P, Lesmana M, Subekti D, *et al.* Antimicrobial resistance of bacterial pathogens associated with diarrheal patients in Indonesia. *Am J Trop Med Hyg* 2003; **68**: 666–70.

97 Tjoa E, Moehario LH, Rukmana A, Rohsiswatmo R. Acinetobacter baumannii: Role in blood stream infection in Neonatal Unit, Dr. Cipto Mangunkusumo Hospital, Jakarta, Indonesia. *Int J Microbiol* 2013; **2013**. DOI:10.1155/2013/180763.

98 Wang H, Chen M, Xu Y, *et al.* Antimicrobial susceptibility of bacterial pathogens associated with community-acquired respiratory tract infections in Asia: Report from the Community-Acquired Respiratory Tract Infection Pathogen Surveillance (CARTIPS) study, 2009-2010. *Int J Antimicrob Agents* 2011; **38**: 376–83.

99 Wardhana A, Djan R, Halim Z. Bacterial and antimicrobial susceptibility profile and the prevalence of sepsis among burn patients at the Burn Unit of Cipto Mangunkusumo Hospital. *Ann Burns Fire Disasters* 2017; **30**: 107–15.

100 Wijaksana DS, Anggraeni N, Endriani R. Pola Bakteri dan Resistensi Antibiotik pada Pasien Sepsis di Intensive Care Unit (ICU) RSUD Arifin Achmad Provinsi Riau Periode 1 Januari – 31 Desember 2017. *J Ilmu Kedokt* 2019; **13**: 46–54.

101 Wikaningtyas P, Sigit J, Sukandar E, Gunawan I. Profile of antibiotic resistance and usage pattern in ICU of private hospital in Bandung, Indonesia. *Int J Pharm Pharm Sci* 2015; **7**: 160–2.

102 Yanagi D, de Vries GC, Rahardjo D, *et al.* Emergence of fluoroquinolone-resistant strains of Salmonella enterica in Surabaya, Indonesia. *Diagn Microbiol Infect Dis* 2009; **64**: 422–6.
